# On analysis of iron (II) chloride via graph entropy measures and statistical models

**DOI:** 10.1371/journal.pone.0294580

**Published:** 2024-01-02

**Authors:** Hao Zhou, Muhammad Farhan Hanif, Hasan Mahmood, Muhammad Kamran Siddiqui, Mazhar Hussain, Samuel Asefa Fufa

**Affiliations:** 1 School of Naval Architecture, Ocean and Energy Power Engineering, Wuhan University of Technology, Wuhan, China; 2 Department of Mathematics and Statistics, The University of Lahore, Lahore Campus, Lahore, Pakistan; 3 Department of Mathematics, Government College University, Lahore, Pakistan; 4 Department of Mathematics, COMSATS University Islamabad, Lahore Campus, Islamabad, Pakistan; 5 Department of Mathematics, Addis Ababa University, Addis Ababa, Ethiopia; VIT University, INDIA

## Abstract

The crystalline material that is greenish-white and dissolves in water is iron chloride. It is utilized in sewage treatment, dyeing, and medicine. Graph entropy plays a significant role in measuring the complexity of atoms, molecules, and structures in nature. It has specific chemical applications in biology, neuroscience, and chemistry. A compound’s molecular structure consists of many atoms. Particularly, hydrocarbons are a chemical combination of hydrogen and carbon atoms. In this article, we discuss the entropy of the chemical structure Iron (II) Chloride. Additionally, we discuss the idea of degree-based indices and compute the Shannon entropy(*ENT*) using these indices. The linear regression(LR) of various indices and entropies for iron chloride, *FeCl*_2_, is also discussed. Also, we link the degree-based indices and entropies via line fit.

## 1. Introduction

A subfield of mathematical chemistry known as chemical graph theory makes use of graph theory to examine and comprehend chemical phenomena. Researchers can efficiently model and research complicated molecule structures and their properties by using graph theory techniques. Chemical graph theory uses a variety of graph theoretical principles and methods to solve molecular issues and shed light on chemical behavior [1].

Let’s have a look at a graph with the symbol *K*, where *V*(*K*) stands for the set of vertices and *E*(*K*) stands for the set of edges. The length of the shortest path within graph *K* linking two vertices *x* and *y* in this context relates to their distance. In addition, the number of nearby edges in *K* is used to define a vertex degree *Q*_*x*_ for a vertex *x*. The order and size of the graph *K* can be used to further describe it. The order of the graph, denoted as *e*, indicates how many vertices there are, while the size of the graph, denoted as *f*, indicates how many edges there are. As a result, a graph *K* is known as a (*e*, *f*) graph when it includes *e* vertices and *f* edges [2].

Based on the molecular network or structure of a material, a topological index is a numerical number that may be determined analytically [3]. It is utilized to build a connection between a substance’s structural features and different physical qualities, chemical reactivity, or biological activity connected to that substance [4]. Zhang et. al [5, 6] discuss the topological indices of generalized bridge molecular graphs, Carbon Nanotubes, and products of chemical graphs. Zhang et. al [7, 8] provided the physical analysis of heat for the formation and entropy of Ceria Oxide. Zhang et. al [9] gives an analysis of different Molecular Structures using Topological indices. The underlying connectivity and configuration of the atoms in a molecule are elucidated by topological indices [10]. By quantifying specific structural traits or patterns, topological indices enable the classification and comparison of molecules, the prediction of their properties, and the investigation of structure-activity relationships. Because they make it simpler to investigate molecular structures and how they affect the behavior and properties of chemical compounds, these indices are crucial for chemical and pharmaceutical research.

Shannon first introduced the idea of entropy in his widely read paper [11], where he described it as a way to quantify uncertainty in a probability distribution. Since then, entropy has been used extensively in the study of chemical networks and graph structures. In the study of graph theory and chemical network analysis, it is now widely used. Measures of graph entropy include a variety of distinct forms, including [12]. For instance, there are intrinsic and extrinsic entropy measures that show relationships between probability distributions and graph building blocks (such as vertices and edges).

Shannon’s groundbreaking work in the late 1940s [11] profoundly changed modern information theory. Information theory was widely used in biology and chemistry as well, despite being initially employed in disciplines like linguistics and electrical engineering, as seen in [13]. According to [14], graph entropy measures have also found wide use in biology, computer science, and structural chemistry. These entropic network metrics have several uses, from studying biological and chemical aspects of molecular graphs to quantitatively characterizing the structure of chemical compounds and software systems. Graph entropy, first proposed by Rashevsky, provides a means to assess the structural complexity of graphs [15]. In Table 1, different topological indices are presented, offering distinct perspectives on graph properties. Throughout this paper, several notations have been developed to facilitate the discussion and understanding of the presented concepts and ideas.

**Table 1:** Degree based topological indices *FeCl*_2_.

TI	Sign	Formula
Randic [16, 17]	*R*_*γ*_(*K*)	$\sum_{xy\in E(K)}(u^{*}{)}^{\gamma };\gamma =1,-1,\frac{1}{2},\frac{-1}{2}$
Atom Bond Connectivity [18]	*ABC*(*K*)	$\sum_{xy\in E(K)}\sqrt{\frac{u^{*}-2}{u^{+}}}$
Geometric Arithimetic [18]	*GA*(*K*)	$\sum_{xy\in E(K)}(\frac{2\sqrt{u^{*}}}{u^{+}})$
First Zagreb [19]	*M*_1_(*K*)	∑_*xy*∈*E*(*K*)_(*u*^+^)
Second Zagreb [20]	*M*_2_(*K*)	∑_*xy*∈*E*(*K*)_(u*)
Hyper Zagreb [21]	*HM*(*K*)	∑_*xy*∈*E*(*K*)_(*u*^+^)^2^
Forgotten [22]	*F*(*K*)	$\sum_{xy\in E(K)}(Q_{x}^{2}+Q_{y}^{2})$
First Redefined Zagreb [22]	*ReZG*_1_(*K*)	$\sum_{xy\in E(K)}(\frac{u^{+}}{u^{*}})$
Second Redefined Zagreb [22]	*ReZG*_2_(*K*)	$\sum_{xy\in E(K)}(\frac{u^{*}}{u^{+}})$
Third Redefined Zagreb [22]	*ReZG*_3_(*K*)	∑_*xy*∈*E*(*K*)_((u*)(*u*^+^))

Consider a simple graph *FeCl*_2_ = *K*(*E*(*K*), *V*(*K*)), where *V*(*K*) be the vertex set of graph *K*, *E*(*K*) be the edge set of graph *K*. We consider *u*^+^ = *Q*_*x*_ + *Q*_*y*_ and u* = *Q*_*x*_ × *Q*_*y*_ in the paper.

**Fig 1:**
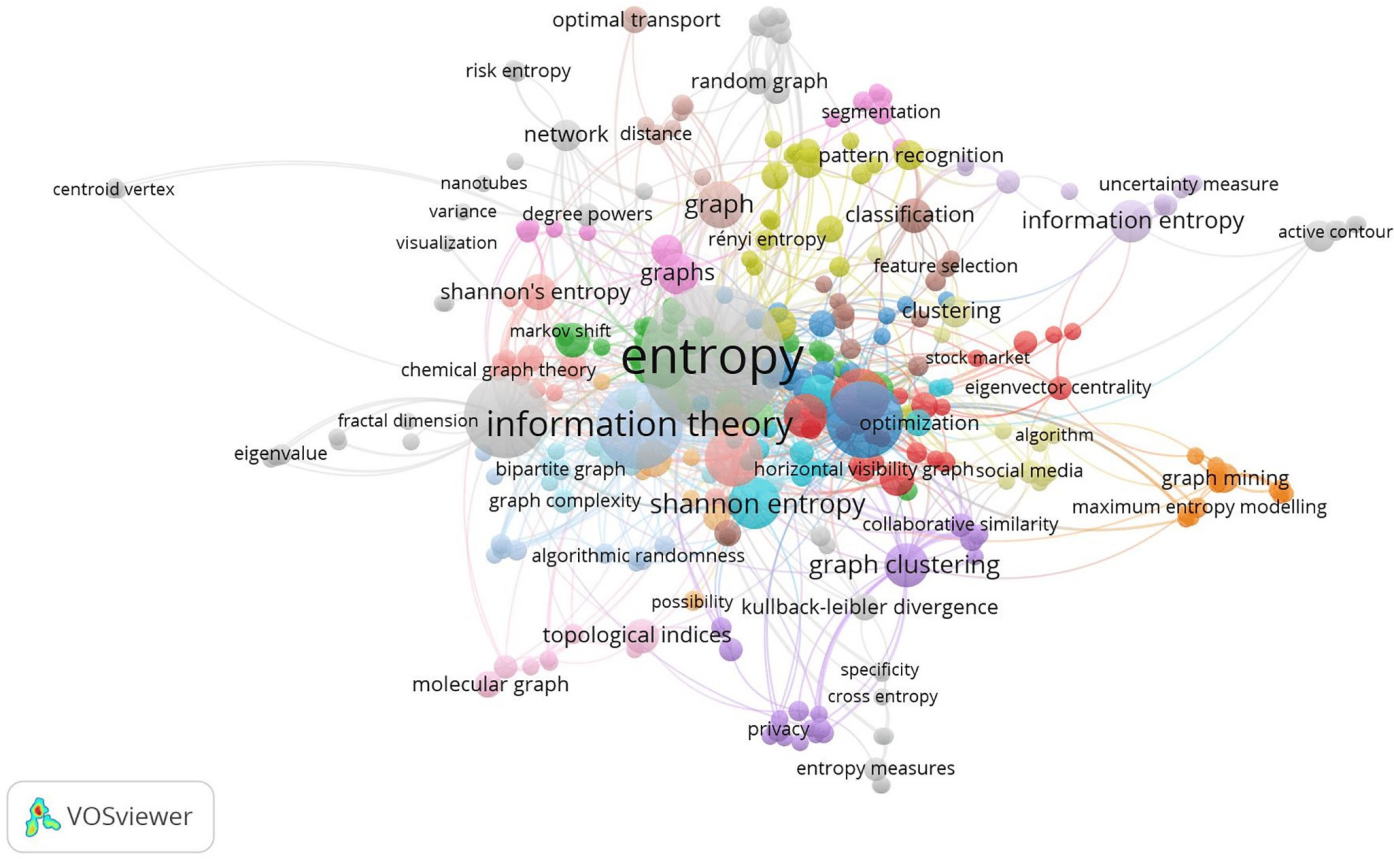
Author-wise bibliometric analysis based on entropy.

The Scopus database (www.scopus.com) was used to conduct the bibliometric analysis of keyword entropy. Fig 1 displays the findings of this investigation and numerous terms associated with entropy. Each keyword is displayed within a unique cluster that is identified by a particular color. Notably, “entropy” appears in the largest circle, indicating that it is the term that has been the subject of the most searches and research. The keywords “Shannon entropy” and “Information theory” are shown in separate circles, indicating their prominence as the second most extensively studied topics. In the larger context of entropy study, the remaining keywords in the graph, such as “graph mining” and “topological indices,” suggest further topics of interest. This bibliometric analysis offers insightful data on the relative popularity and research emphasis with different entropy-related keywords.

**Fig 2:**
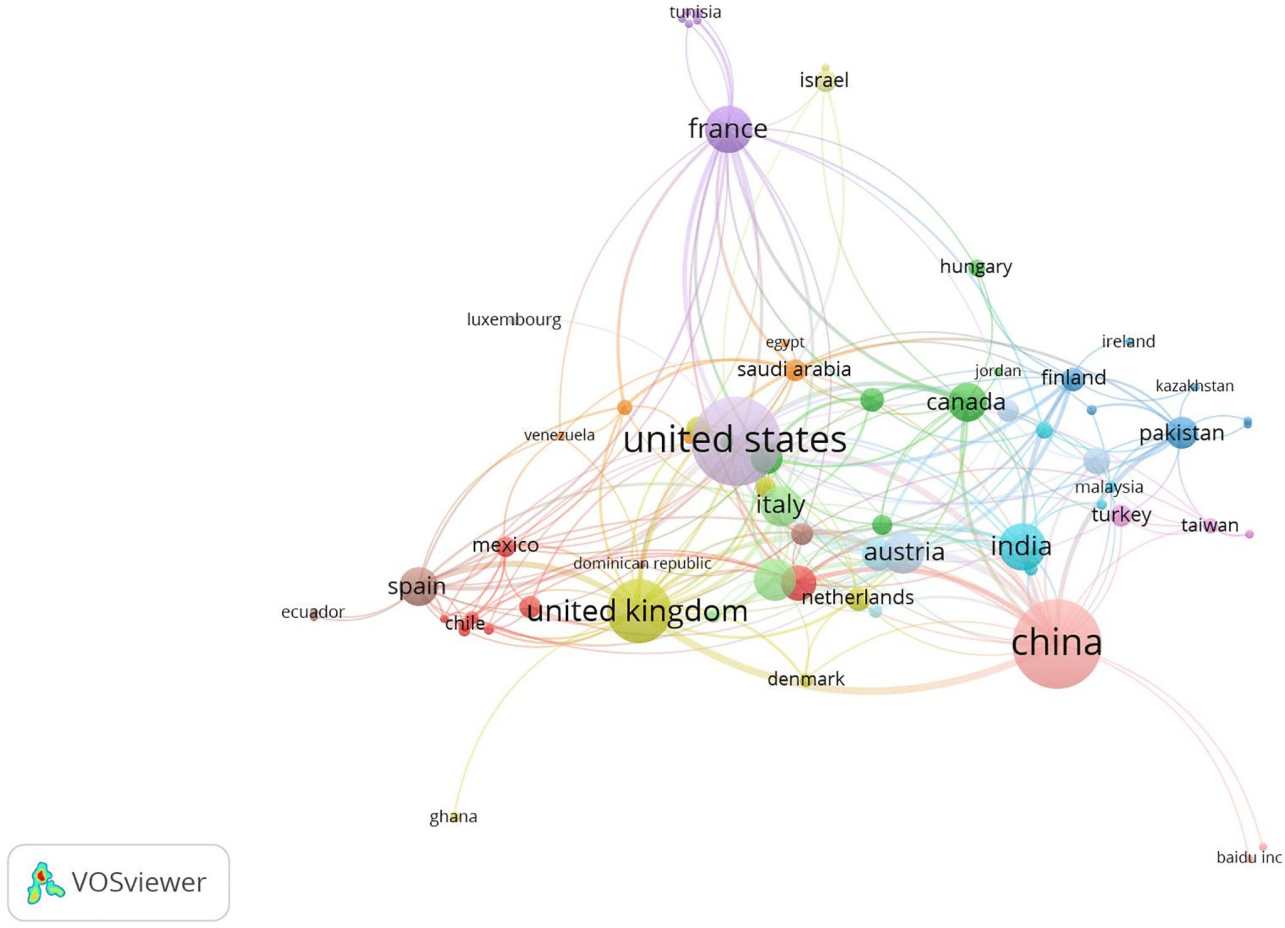
Bibliometric analysis of country-wise research based on entropy.

The Scopus database (www.scopus.com) was used to conduct the bibliometric analysis of entropy-related research in various nations. The results of this investigation are shown in Fig 2, which also indicates which nations are leading in entropy research. With a significant number of scholars actively involved in entropy-related studies, the United States emerges as the major contributor in the field. In addition to the United States, Pakistan, and India have considerable entropy research efforts, demonstrating their expanding involvement and interest in the field. Other nations, albeit to a lesser level, make significant contributions to entropy research, such as the United Kingdom, Saudi Arabia, Austria, and Spain. This bibliometric analysis sheds light on the geographic distribution of research activities and the relative importance of various nations in the study of entropy.

In 2014, a revolutionary concept regarding the entropy of an edge-weighted graph was developed in a groundbreaking work by Chen et al. [23, 24]. Examining an Iron II Chloride graph, represented as *FeCl*_2_ = *K* = (*V*(*K*); *E*(*K*); *ψ*(*αβ*)), in which *V*(*K*) stands for the vertices, *E*(*K*) for the edges, and *ψ*(*αβ*) for the weight assigned to each edge *ψ*(*αβ*) in *K*, was the main objective. Eq 1 expresses the researcher’s concept for calculating the edge-weighted graph entropy. This method sought to provide a full comprehension of the information content and the complex structure of the graph. Edge-weighted graph entropy theory brought up fresh perspectives on the properties and patterns that these systems naturally possess.

$$\begin{array}{c} ENT_{\psi }\left(K\right)=-\sum_{\alpha^{\prime }\beta^{\prime }\in E\left(K\right)}\frac{\psi (\alpha^{\prime }\beta^{\prime })}{\sum_{xy\in E(K)}\psi \left(\alpha \beta \right)}\;\text{log}\;[\frac{\alpha (\phi^{\prime }\beta^{\prime })}{\sum_{\alpha \beta \in E(K)}\psi \left(\alpha \beta \right)}] \end{array}$$ (1)

Using the different indices in Eq 1 we get the following Table 2.

**Table 2:** Entropy for different degree based indices *FeCl*_2_.

*ENT*	Sign	Formula
Randic	*ER*_*γ*_(*K*)	$\text{log}(R_{\gamma })-\frac{1}{\left(R_{\gamma }\right)}\sum_{i=1}^{m}\sum_{xy\in E_{i}(K)}(u^{*}{)}^{\gamma }\;\text{log}\;(u^{*}{)}^{\gamma };\gamma =1,-1,\frac{1}{2},\frac{-1}{2}$
Atom Bond Connectivity	*EABC*(*K*)	$\text{log}(ABC)-\frac{1}{\left(ABC\right)}\sum_{i=1}^{m}\sum_{xy\in E_{i}(K)}(\sqrt{\frac{u^{+}-2}{u^{*}}})\;\text{log}\;\left(\sqrt{\frac{u^{+}-2}{u^{*}}}\right)$
Geometric Arithmetic	*EGA*(*K*)	$\text{log}(GA)-\frac{1}{\left(GA\right)}\sum_{i=1}^{m}\sum_{xy\in E_{i}(K)}(\frac{2\sqrt{u^{*}}}{u^{+}})\;\text{log}\;\left(\frac{2\sqrt{u^{*}}}{u^{+}}\right)$
First Zagreb	*EM*_1_(*K*)	$\text{log}(M_{1})-\frac{1}{\left(M_{1}\right)}\sum_{i=1}^{m}\sum_{xy\in E_{i}(K)}(u^{+})\;\text{log}\;\left(u^{+}\right)$
Second Zagreb	*EM*_2_(*K*)	$\text{log}(M_{1})-\frac{1}{\left(M_{2}\right)}\sum_{i=1}^{m}\sum_{xy\in E_{i}(K)}(u^{*})\;\text{log}\;\left(u^{*}\right)$
Hyper Zagreb	*EHM*(*K*)	$\text{log}(HM)-\frac{1}{\left(HM\right)}\sum_{i=1}^{m}\sum_{xy\in E_{i}(K)}((u^{+}{)}^{2})\;\text{log}\;\left((u^{+}{)}^{2}\right)$
Forgotten	*EF*(*K*)	$\text{log}(F)-\frac{1}{\left(F\right)}\sum_{i=1}^{m}\sum_{xy\in E_{i}(K)}((Q_{x}{)}^{2}+(Q_{y}{)}^{2})\;\text{log}\;\left((Q_{x}{)}^{2}+(Q_{y}{)}^{2}\right)$
First Redefined Zagreb	*EReZG*_1_(*K*)	$\text{log}(ReZG_{1})-\frac{1}{\left(ReZG_{1}\right)}\sum_{i=1}^{m}\sum_{xy\in E_{i}(K)}(\frac{u^{+}}{u^{*}})\;\text{log}\;\left(\frac{u^{+}}{u^{*}}\right)$
Second Redefined Zagreb	*EReZG*_2_(*K*)	$\text{log}(ReZG_{2})-\frac{1}{\left(ReZG_{2}\right)}\sum_{i=1}^{m}\sum_{xy\in E_{i}(K)}(\frac{u^{*}}{u^{+}})\;\text{log}\;\left(\frac{u^{*}}{u^{+}}\right)$
Third Redefined Zagreb	*EReZG*_3_(*K*)	$\text{log}(ReZG_{3})-\frac{1}{\left(ReZG_{3}\right)}\sum_{i=1}^{m}\sum_{xy\in E_{i}(K)}(\left(u^{*}\right)\times \left(u^{+}\right))\;\text{log}\;\left(\left(u^{*}\right)\times \left(u^{+}\right)\right)$

## 2. Research aim and methodology

Our purpose in this paper is to discuss the chemical graph of Iron II Chloride crystalline structure. We compute the indices of the Iron II Chloride crystalline structure namely, Randic indices, Atom bond connectivity index, etc. Also, we compute the entropies of these structures by using the above-mentioned indices.

The methodology of this paper is as follows: In section 3 we discuss the crystalline structure of Iron II Chloride. In section 4 we compute the entropy by using the topological indices. In section 5 we discuss the correlation between indices and entropy values. In section 6 we present the conclusion of our paper.

## 3. Iron (II) chloride structure

Iron (II) chloride, also known as ferrous chloride, is a white inorganic salt of iron and chlorine with the chemical formula *FeCl*_2_. Magnetically it shows paramagnetic behavior and it has a high melting point as it is solid with strong chemical bonding. Simple iron (II) chloride is rarely used; its hydrated forms are commonly encountered in industrial and laboratory applications. Tetrahydrate of iron (II) chloride is commonly used in industry and it also exists in the form of dehydrated. This salt is highly soluble in water and it gives a green color solution [25, 26]. Wold et al. [27] Synthesized iron (II) chloride through the reaction of iron with hydrochloric acid in nitrogen inert environment. In a typical procedure, iron powder and methanol were added to a vessel followed by the addition of hydrochloric acid. To provide an inert environment and prevent diffusion of atmospheric oxygen a gas curtain of nitrogen was employed in the reaction. A vessel containing the reaction mixture was placed in hot water for 2–3 hours till the completion of the reaction. The formation of a greenish-gray solution indicates the formation of methylated iron (II) chloride; to remove extra solvent product was dried in multiple steps at different temperature conditions.

**Fig 3:**
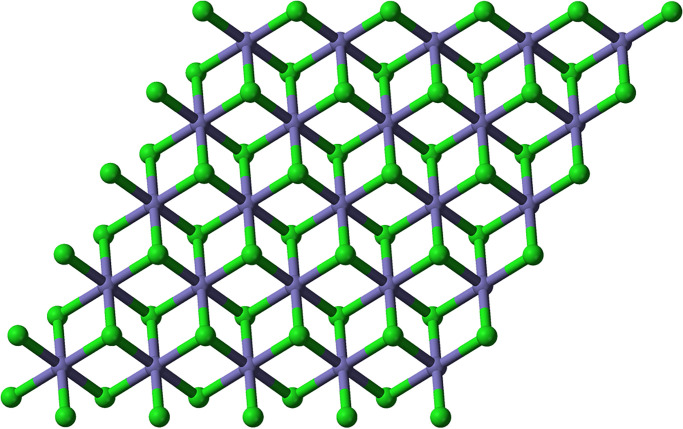
Iron(II) chloride *FeCl*_2_ for n = 1.

**Fig 4:**
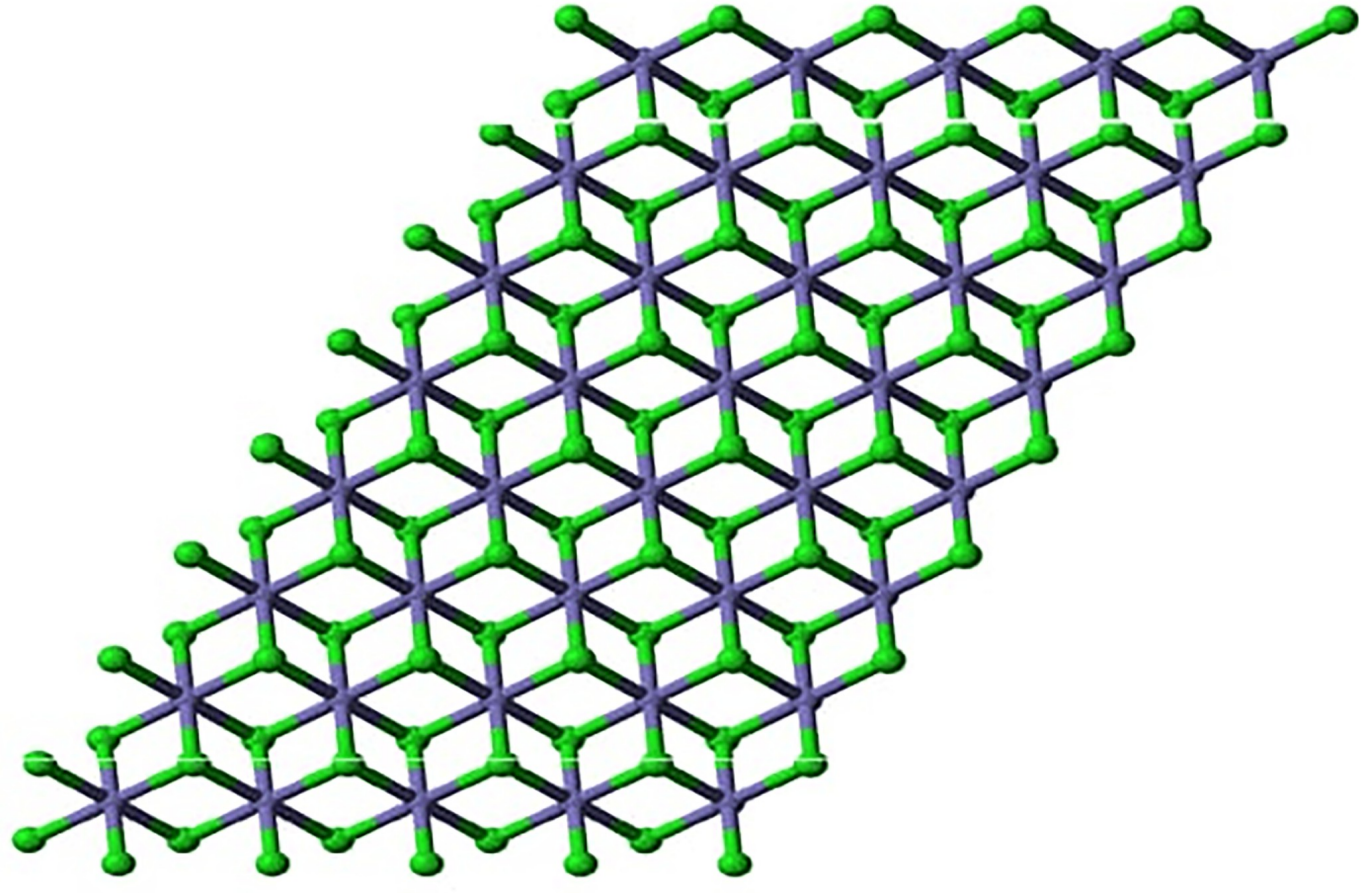
Iron(II) chloride *FeCl*_2_ for n = 2.

**Fig 5:**
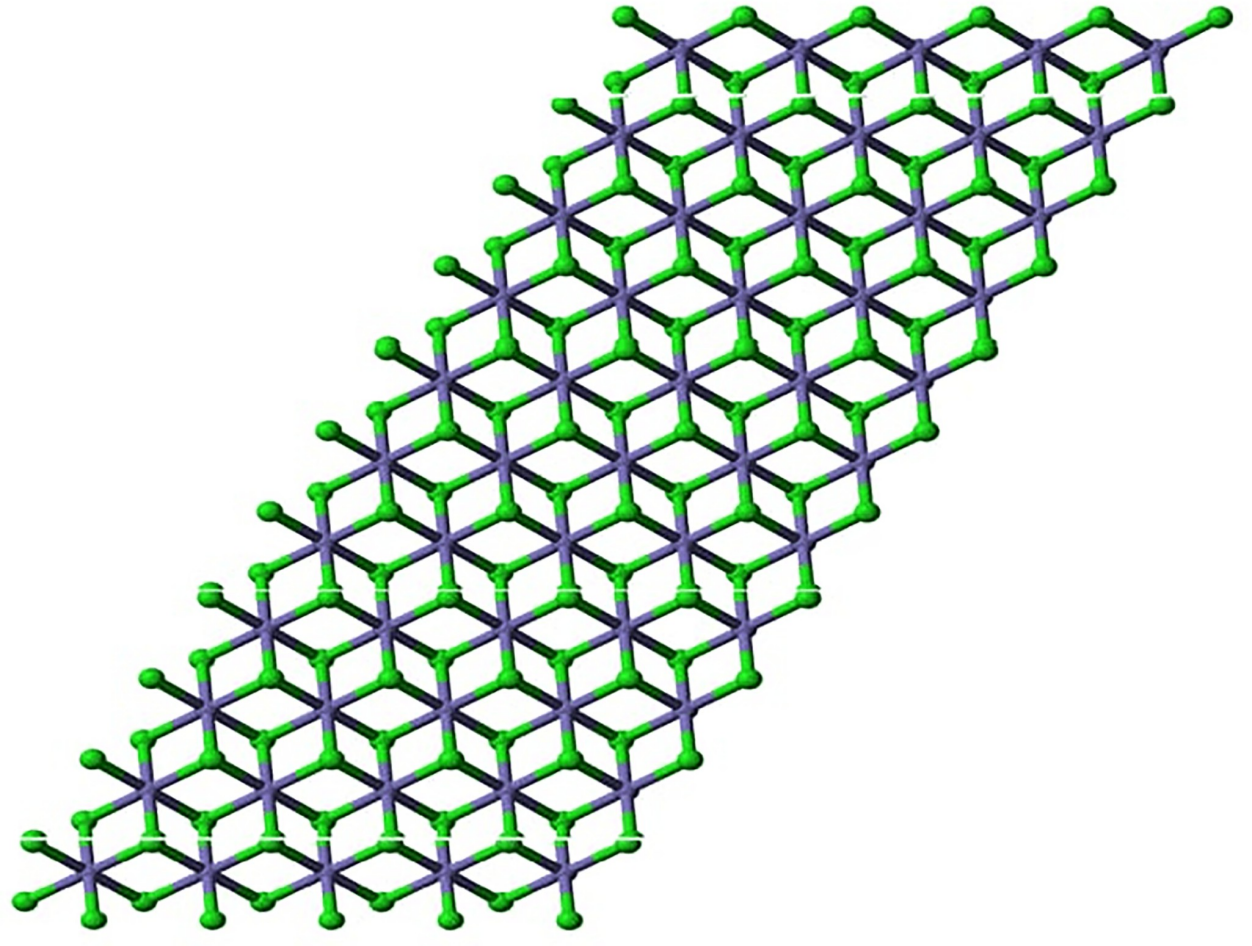
Iron(II) chloride *FeCl*_2_ for n = 3.

In another simple method for the preparation of iron (II) chloride chlorobenzene was refluxed with anhydrous ferric chloride for 220 minutes, produce was washed with anhydrous benzene and the reported yield was 97% [28]. Tetrahydrofuran compounds of iron(II) chloride have been employed to elaborate its tetrahedral and octahedral structure with different ligands, and a simple ball and stick crystalline model of iron(II) chloride has been presented in the following figure [29, 30]. Iron (II) chloride reacts with several organic and inorganic ligands to produce a variety of complexes; it has been used to synthesize nitrogenous complexes and complexes with hydrogen peroxide. Organic complexes with tetra hydro furan have been considered precursors to many iron-based organometallic compounds [31]. Cross-coupling reactions are the most important reactions of synthetic organic chemistry both in terms of synthesis and mechanism; iron(II) chloride has been used to catalyze these cross-coupling reactions [32]. Green rust is the important intermediate substance of oxidized iron that leads to corrosion; it is formed by the oxidation of iron in the presence of chlorine and iron (II) chloride is one of the major compounds that is formed in green rust [33]. Unlike other iron compounds, iron (II) chloride is only limitedly used; in addition to synthesizing iron complexes in the laboratory, they are used in wastewater treatment as flocculation and coagulation agents. Reportedly, it has been employed for the coagulation and flocculation of chromates and sulfates in wastewater as an efficient procedure aimed at wastewater treatment. The structure of Iron (II) chloride is shown in Fig 3 for n = 1, Fig 4 for n = 2, and Fig 5 for n = 3.

Different crystalline configurations of iron chloride (*FeCl*_2_) can be observed depending on the temperature and pressure of the surrounding air. It adopts a crystal structure known as the “rutile” or “distorted rutile” structure under normal ambient temperature and pressure [34]. In Fig 1, *n* represents the one unit of distorted rutile structure. In one unit, each iron (Fe) atom (blue color) in the rutile configuration is surrounded by six chloride (Cl) atoms (green color), with three iron atoms surrounding each chloride atom [35]. The chloride ions are arranged in a deformed hexagonal close-packed (hcp) lattice, and the iron ions adopt octahedral coordination with them [36].

The size of the graph is 87*n* + 53. The edge partition is shown in Table 3.

**Table 3:** Edge partition of iron (II) chloride *FeCl*_2_.

(*Q*_*x*_, *Q*_*y*_)	$\left|E_{i(FeCl_{2})}\right|$
(1, 4)	|*E*_1_| = 1
(1, 5)	|*E*_2_| = 2
(1, 6)	|*E*_3_| = 3*n* + 6
(2, 4)	|*E*_4_| = 2
(2, 5)	|*E*_5_| = 6*n* + 10
(2, 6)	|*E*_6_| = 6*n* + 8
(3, 4)	|*E*_7_| = 1
(3, 5)	|*E*_8_| = 9*n* + 13
(3, 6)	|*E*_9_| = 63*n* + 10

## 4. Computing topological index based entropy and numerical indices values

In this section, we do topological indices-based entropy computations. The entropy values connected to particular topological indices are calculated using mathematical formulas and algorithms. We can quantify and assess the complexity and information content of different chemical structures using these methods. An in-depth understanding of the features and behavior of molecules is made possible by the analysis of topological indices-based entropy, which offers insightful information about the structural qualities and connectivity patterns of molecules.

- **Randic entropy of** *FeCl*_2_

Using Tables 1 and 3 the Randic index for $\gamma =1,-1,\frac{1}{2},\frac{-1}{2}$ is:

$$\begin{array}{ccc} R_{1}\left(FeCl_{2}\right) & = & {\left(4\right)}^{\gamma }\left(1\right)+{\left(5\right)}^{\gamma }\left(2\right)+{\left(6\right)}^{\gamma }\left(3n+6\right)+{\left(8\right)}^{\gamma }\left(2\right)+{\left(10\right)}^{\gamma }\left(6n+10\right)\\ & + & {\left(12\right)}^{\gamma }\left(6n+8\right)+{\left(12\right)}^{\gamma }\left(1\right)+{\left(15\right)}^{\gamma }\left(9n+13\right)+{\left(18\right)}^{\gamma }\left(63n+10\right) \end{array}$$

Using Tables 1–3 we have the following equation because *FeCl*_2_ has nine types of edges:

$$\begin{array}{ccc} ENT_{R_{\gamma }} & = & \text{log}\left(R_{\gamma }\right)-\frac{1}{\left(R_{\gamma }\right)}\sum_{i=1}^{9}\sum_{xy\in E_{i}\left(K\right)}\left(u^{*}\right)\;\text{log}\;\left(u^{*}\right) \end{array}$$

After putting value of Randic index for $\gamma =1,-1,\frac{1}{2},\frac{-1}{2}$ and using Table 3 we have.

$$\begin{array}{ccc} ENT_{R_{1}} & = & \text{log}\left(1419n+649\right)-\frac{\left(1\right)\;\text{log}({\left(4\right)}^{4})}{\left(1419n+649\right)}-\frac{\left(2\right)\;\text{log}({\left(5\right)}^{5})}{\left(1419n+649\right)}-\frac{\left(3n+6\right)\;\text{log}({\left(6\right)}^{6})}{\left(1419n+649\right)}\\ & - & \frac{\left(2\right)\;\text{log}({\left(8\right)}^{8})}{\left(1419n+649\right)}-\frac{\left(6n+10\right)\;\text{log}({\left(10\right)}^{10})}{\left(1419n+649\right)}-\frac{\left(6n+8\right)\;\text{log}({\left(12\right)}^{12})}{\left(1419n+649\right)}\\ & - & \frac{\left(1\right)\;\text{log}({\left(12\right)}^{12})}{\left(1419n+649\right)}-\frac{\left(9n+3\right)\;\text{log}({\left(15\right)}^{15})}{\left(1419n+649\right)}-\frac{\left(63n+10\right)\;\text{log}({\left(18\right)}^{18})}{\left(1419n+649\right)} \end{array}$$

$$\begin{array}{ccc} ENT_{R_{-1}} & = & \text{log}\left(5.7n+5.0722\right)-\frac{\left(1\right)\;\text{log}({\left(4\right)}^{-\frac{1}{4}})}{\left(5.7n+5.0722\right)}-\frac{\left(2\right)\;\text{log}({\left(5\right)}^{-\frac{1}{5}})}{\left(5.7n+5.0722\right)}\\ & - & \frac{\left(3n+6\right)\;\text{log}({\left(6\right)}^{-\frac{1}{6}})}{\left(5.7n+5.0722\right)}\\ & - & \frac{\left(2\right)\;\text{log}({\left(8\right)}^{-\frac{1}{8}})}{\left(5.7n+5.0722\right)}-\frac{\left(6n+10\right)\;\text{log}({\left(10\right)}^{-\frac{1}{10}})}{\left(5.7n+5.0722\right)}-\frac{\left(6n+8\right)\;\text{log}({\left(12\right)}^{-\frac{1}{12}})}{\left(5.7n+5.0722\right)}\\ & - & \frac{\left(1\right)\;\text{log}({\left(12\right)}^{-\frac{1}{12}})}{\left(5.7n+5.0722\right)}-\frac{\left(9n+3\right)\;\text{log}({\left(15\right)}^{-\frac{1}{15}})}{\left(5.7n+5.0722\right)}-\frac{\left(63n+10\right)\;\text{log}({\left(18\right)}^{-\frac{1}{18}})}{\left(5.7n+5.0722\right)} \end{array}$$

$$\begin{array}{ccc} ENT_{R_{\frac{1}{2}}} & = & \text{log}\left(349.2499n+182.4008\right)-\frac{\left(1\right)\;\text{log}({\left(4\right)}^{\frac{\sqrt{4}}{2}})}{\left(349.2499n+182.4008\right)}\\ & - & \frac{\left(2\right)\;\text{log}({\left(5\right)}^{\frac{\sqrt{5}}{2}})}{\left(349.2499n+182.4008\right)}-\frac{\left(3n+6\right)\;\text{log}({\left(6\right)}^{\frac{\sqrt{6}}{2}})}{\left(349.2499n+182.4008\right)}\\ & - & \frac{\left(2\right)\;\text{log}({\left(8\right)}^{\frac{\sqrt{8}}{2}})}{\left(349.2499n+182.4008\right)}-\frac{\left(6n+10\right)\;\text{log}({\left(10\right)}^{\frac{\sqrt{10}}{2}})}{\left(349.2499n+182.4008\right)}\\ & - & \frac{\left(6n+8\right)\;\text{log}({\left(12\right)}^{\frac{\sqrt{12}}{2}})}{\left(349.2499n+182.4008\right)}-\frac{\left(1\right)\;\text{log}({\left(12\right)}^{\frac{\sqrt{12}}{2}})}{\left(349.2499n+182.4008\right)}\\ & - & \frac{\left(9n+3\right)\;\text{log}({\left(15\right)}^{\frac{\sqrt{15}}{2}})}{\left(349.2499n+182.4008\right)}-\frac{\left(63n+10\right)\;\text{log}({\left(18\right)}^{\frac{\sqrt{18}}{2}})}{\left(349.2499n+182.4008\right)} \end{array}$$

$$\begin{array}{ccc} ENT_{R_{-\frac{1}{2}}} & = & \text{log}\left(22.0272n+16.0249\right)-\frac{\left(1\right)\;\text{log}({\left(4\right)}^{-\frac{\sqrt{4}}{8}})}{\left(22.0272n+16.0249\right)}\\ & - & \frac{\left(2\right)\;\text{log}({\left(5\right)}^{-\frac{\sqrt{5}}{10}})}{\left(22.0272n+16.0249\right)}-\frac{\left(3n+6\right)\;\text{log}({\left(6\right)}^{-\frac{\sqrt{6}}{12}})}{\left(22.0272n+16.0249\right)}\\ & - & \frac{\left(2\right)\;\text{log}({\left(8\right)}^{-\frac{\sqrt{8}}{16}})}{\left(22.0272n+16.0249\right)}-\frac{\left(6n+10\right)\;\text{log}({\left(10\right)}^{-\frac{\sqrt{10}}{20}})}{\left(22.0272n+16.0249\right)}\\ & - & \frac{\left(6n+8\right)\;\text{log}({\left(12\right)}^{-\frac{\sqrt{12}}{24}})}{\left(22.0272n+16.0249\right)}-\frac{\left(1\right)\;\text{log}({\left(12\right)}^{-\frac{\sqrt{12}}{24}})}{\left(22.0272n+16.0249\right)}\\ & - & \frac{\left(9n+3\right)\;\text{log}({\left(15\right)}^{-\frac{\sqrt{15}}{30}})}{\left(22.0272n+16.0249\right)}-\frac{\left(63n+10\right)\;\text{log}({\left(18\right)}^{-\frac{\sqrt{18}}{36}})}{\left(22.0272n+16.0249\right)} \end{array}$$

- **The Atom Bond Connectivity entropy of** *FeCl*_2_

Using Tables 1 and 3 the computed atom bond connectivity index is:

$$\begin{array}{c} ABC(FeCl_{2})=56.2033n+37.3777. \end{array}$$

Using Tables 1–3 we have the following equation because *FeCl*_2_ has nine types of edges:

$$\begin{array}{c} ENT_{ABC}=\text{log}\left(ABC\right)-\frac{1}{\left(ABC\right)}\sum_{i=1}^{9}\sum_{xy\in E_{i}\left(K\right)}(\sqrt{\frac{u^{+}-2}{u^{*}}})\;\text{log}\;(\sqrt{\frac{u^{+}-2}{u^{*}}}) \end{array}$$

After putting the value of atom bond connectivity index and using Table 3 we get.

$$\begin{array}{ccc} ENT_{ABC} & = & \text{log}\left(56.2033n+37.3777\right)-\frac{\left(1\right)\;\text{log}({\left(\sqrt{\frac{3}{4}}\right)}^{\sqrt{\frac{3}{4}}})}{\left(56.2033n+37.3777\right)}\\ & - & \frac{\left(2\right)\;\text{log}({\left(\sqrt{\frac{4}{5}}\right)}^{\sqrt{\frac{4}{5}}})}{\left(56.2033n+37.3777\right)}-\frac{\left(3n+6\right)\;\text{log}({\left(\sqrt{\frac{5}{6}}\right)}^{\sqrt{\frac{5}{6}}})}{\left(56.2033n+37.3777\right)}\\ & - & \frac{\left(2\right)\;\text{log}({\left(\sqrt{\frac{4}{8}}\right)}^{\sqrt{\frac{4}{8}}})}{\left(56.2033n+37.3777\right)}-\frac{\left(6n+10\right)\;\text{log}({\left(\sqrt{\frac{5}{10}}\right)}^{\sqrt{\frac{5}{10}}})}{\left(56.2033n+37.3777\right)}\\ & - & \frac{\left(6n+8\right)\;\text{log}({\left(\sqrt{\frac{6}{12}}\right)}^{\sqrt{\frac{6}{12}}})}{\left(56.2033n+37.3777\right)}-\frac{\left(1\right)\;\text{log}({\left(\sqrt{\frac{5}{12}}\right)}^{\sqrt{\frac{5}{12}}})}{\left(56.2033n+37.3777\right)}\\ & - & -\frac{\left(9n+3\right)\;\text{log}({\left(\sqrt{\frac{6}{15}}\right)}^{\sqrt{\frac{6}{15}}})}{\left(56.2033n+37.3777\right)}-\frac{\left(63n+10\right)\;\text{log}({\left(\sqrt{\frac{7}{18}}\right)}^{\sqrt{\frac{7}{18}}})}{\left(56.2033n+37.3777\right)} \end{array}$$

- **The Geometric Arithmetic entropy of** *FeCl*_2_

Using Tables 1 and 3 the computed geometric arithmetic index is:

$$\begin{array}{c} GA(FeCl_{2})=80.8279n+47.3438. \end{array}$$

Using Tables 1–3 we have the following equation because *FeCl*_2_ has nine types of edges:

$$\begin{array}{c} ENT_{GA}\left(K\right)=\text{log}\left(GA\right)-\frac{1}{\left(GA\right)}\sum_{i=1}^{9}\sum_{xy\in E_{i}\left(K\right)}(\frac{2\sqrt{u^{*}}}{u^{+}})\;\text{log}\;(\frac{2\sqrt{u^{*}}}{u^{+}}) \end{array}$$

After putting value of geometric arithmetic index and using Table 3 we get.

$$\begin{array}{ccc} ENT_{GA} & = & \text{log}\left(80.8279n+47.3438\right)-\frac{\left(1\right)\;\text{log}({\left(\frac{2\sqrt{4}}{5}\right)}^{\frac{2\sqrt{4}}{5}})}{\left(80.8279n+47.3438\right)}\\ & - & \frac{\left(2\right)\;\text{log}({\left(\frac{2\sqrt{5}}{6}\right)}^{\frac{2\sqrt{5}}{6}})}{\left(80.8279n+47.3438\right)}-\frac{\left(3n+6\right)\;\text{log}({\left(\frac{2\sqrt{6}}{7}\right)}^{\frac{2\sqrt{6}}{7}})}{\left(80.8279n+47.3438\right)}\\ & - & \frac{\left(2\right)\;\text{log}({\left(\frac{2\sqrt{8}}{6}\right)}^{\frac{2\sqrt{8}}{6}})}{\left(80.8279n+47.3438\right)}-\frac{\left(6n+10\right)\;\text{log}({\left(\frac{2\sqrt{10}}{7}\right)}^{\frac{2\sqrt{10}}{7}})}{\left(80.8279n+47.3438\right)}\\ & - & \frac{\left(6n+8\right)\;\text{log}({\left(\frac{2\sqrt{12}}{8}\right)}^{\frac{2\sqrt{12}}{8}})}{\left(80.8279n+47.3438\right)}-\frac{\left(1\right)\;\text{log}({\left(\frac{2\sqrt{12}}{7}\right)}^{\frac{2\sqrt{12}}{7}})}{\left(80.8279n+47.3438\right)}\\ & - & -\frac{\left(9n+3\right)\;\text{log}({\left(\frac{2\sqrt{15}}{8}\right)}^{\frac{2\sqrt{15}}{8}})}{\left(80.8279n+47.3438\right)}-\frac{\left(63n+10\right)\;\text{log}({\left(\frac{2\sqrt{18}}{9}\right)}^{\frac{2\sqrt{18}}{9}})}{\left(80.8279n+47.3438\right)} \end{array}$$

- **First Zagreb entropy of** *FeCl*_2_

Using Tables 1 and 3 the computed First Zagreb index is:

$$\begin{array}{c} M_{1}\left(FeCl_{2}\right)=750n+406. \end{array}$$

Using Tables 1–3 we have the following equation because *FeCl*_2_ has nine types of edges:

$$\begin{array}{c} ENT_{M_{1}}=\text{log}\left(M_{1}\right)-\frac{1}{\left(M_{1}\right)}\sum_{i=1}^{9}\sum_{xy\in E_{i}\left(K\right)}\left(u^{+}\right)\;\text{log}\;\left(u^{+}\right) \end{array}$$

After putting value of first Zagreb index and using Table 3 we get.

$$\begin{array}{ccc} ENT_{M_{1}} & = & \text{log}\left(750n+406\right)-\frac{\left(1\right)\;\text{log}({\left(5\right)}^{5})}{\left(750n+406\right)}-\frac{\left(2\right)\;\text{log}({\left(6\right)}^{6})}{\left(750n+406\right)}-\frac{\left(3n+6\right)\;\text{log}({\left(7\right)}^{7})}{\left(750n+406\right)}\\ & - & \frac{\left(2\right)\;\text{log}({\left(6\right)}^{6})}{\left(750n+406\right)}-\frac{\left(6n+10\right)\;\text{log}({\left(7\right)}^{7})}{\left(750n+406\right)}-\frac{\left(6n+8\right)\;\text{log}({\left(8\right)}^{8})}{\left(750n+406\right)}-\frac{\left(1\right)\;\text{log}({\left(7\right)}^{7})}{\left(750n+406\right)}\\ & - & \frac{\left(9n+3\right)\;\text{log}({\left(8\right)}^{8})}{\left(750n+406\right)}-\frac{\left(63n+10\right)\;\text{log}({\left(9\right)}^{9})}{\left(750n+406\right)} \end{array}$$

- **Second Zagreb entropy of** *FeCl*_2_

Using Tables 1 and 3 the computed second Zagreb index is:

$$\begin{array}{c} M_{2}\left(FeCl_{2}\right)=1419n+649. \end{array}$$

Using Tables 1–3 we have the following equation because *FeCl*_2_ has nine types of edges:

$$\begin{array}{c} ENT_{M_{2}}=\text{log}\left(M_{1}\right)-\frac{1}{\left(M_{2}\right)}\sum_{i=1}^{m}\sum_{xy\in E_{i}\left(K\right)}\left(u^{*}\right)\;\text{log}\;\left(u^{*}\right) \end{array}$$

After putting value of Second Zagreb index and using Table 3 we get.

$$\begin{array}{ccc} ENT_{M_{2}} & = & \text{log}\left(1419n+649\right)-\frac{\left(1\right)\;\text{log}({\left(4\right)}^{4})}{\left(1419n+649\right)}-\frac{\left(2\right)\;\text{log}({\left(5\right)}^{5})}{\left(1419n+649\right)}-\frac{\left(3n+6\right)\;\text{log}({\left(6\right)}^{6})}{\left(1419n+649\right)}\\ & - & \frac{\left(2\right)\;\text{log}({\left(8\right)}^{8})}{\left(1419n+649\right)}-\frac{\left(6n+10\right)\;\text{log}({\left(10\right)}^{10})}{\left(1419n+649\right)}-\frac{\left(6n+8\right)\;\text{log}({\left(12\right)}^{12})}{\left(1419n+649\right)}\\ & - & \frac{\left(1\right)\;\text{log}({\left(12\right)}^{12})}{\left(1419n+649\right)}\\ & - & \frac{\left(9n+3\right)\;\text{log}({\left(15\right)}^{15})}{\left(1419n+649\right)}-\frac{\left(63n+10\right)\;\text{log}({\left(18\right)}^{18})}{\left(1419n+649\right)} \end{array}$$

- **Hyper Zagreb entropy of** *FeCl*_2_

Using Tables 1 and 3 the computed Hyper Zagreb index is:

$$\begin{array}{c} HM(FeCl_{2})=6504n+3156. \end{array}$$

Using Tables 1–3 we have the following equation because *FeCl*_2_ has nine types of edges:

$$ENT_{HM}=log\left(HM\right)-\frac{1}{\left(HM\right)}\sum_{i=1}^{m}\sum_{xy\in E_{i}\left(K\right)}\left({\left(u^{+}\right)}^{2}\right)\;\text{log}\;\left({\left(u^{+}\right)}^{2}\right)$$

After putting value of Hyper Zagreb index and using Table 3 we get.

$$\begin{array}{ccc} ENT_{HM} & = & \text{log}\left(6504n+3156\right)-\frac{\left(1\right)\;\text{log}({\left(25\right)}^{25})}{\left(6504n+3156\right)}-\frac{\left(2\right)\;\text{log}({\left(36\right)}^{36})}{\left(6504n+3156\right)}\\ & - & \frac{\left(2\right)\;\text{log}({\left(36\right)}^{36})}{\left(6504n+3156\right)}-\frac{\left(6n+10\right)\;\text{log}({\left(49\right)}^{49})}{\left(6504n+3156\right)}-\frac{\left(6n+8\right)\;\text{log}({\left(64\right)}^{64})}{\left(6504n+3156\right)}\\ & - & \frac{\left(9n+3\right)\;\text{log}({\left(64\right)}^{64})}{\left(6504n+3156\right)}-\frac{\left(63n+10\right)\;\text{log}({\left(81\right)}^{81})}{\left(6504n+3156\right)}\\ & - & \frac{\left(3n+6\right)\;\text{log}({\left(49\right)}^{49})}{\left(6504n+3156\right)}-\frac{\left(1\right)\;\text{log}({\left(49\right)}^{49})}{\left(6504n+3156\right)} \end{array}$$

- **Forgotten entropy of** *FeCl*_2_

Using Tables 1 and 3 the computed Forgotten index is:

$$\begin{array}{c} F(FeCl_{2})=3666n+1858. \end{array}$$

Using Tables 1–3 we have the following equation because *FeCl*_2_ has nine types of edges:

$$\begin{array}{c} ENT_{F}=\text{log}\left(F\right)-\frac{1}{\left(F\right)}\sum_{i=1}^{m}\sum_{xy\in E_{i}\left(K\right)}\left({\left(Q_{x}\right)}^{2}+{\left(Q_{y}\right)}^{2}\right)\;\text{log}\;\left({\left(Q_{x}\right)}^{2}+{\left(Q_{y}\right)}^{2}\right) \end{array}$$

After putting value of Forgotten index and using Table 3 we get.

$$\begin{array}{ccc} ENT_{F} & = & \text{log}\left(3666n+1858\right)-\frac{\left(1\right)\;\text{log}({\left(17\right)}^{17})}{\left(3666n+1858\right)}-\frac{\left(2\right)\;\text{log}({\left(26\right)}^{26})}{\left(3666n+1858\right)}\\ & - & \frac{\left(2\right)\;\text{log}({\left(20\right)}^{20})}{\left(3666n+1858\right)}-\frac{\left(6n+10\right)\;\text{log}({\left(29\right)}^{29})}{\left(3666n+1858\right)}-\frac{\left(6n+8\right)\;\text{log}({\left(40\right)}^{40})}{\left(3666n+1858\right)}\\ & - & \frac{\left(9n+3\right)\;\text{log}({\left(34\right)}^{34})}{\left(3666n+1858\right)}-\frac{\left(63n+10\right)\;\text{log}({\left(45\right)}^{45})}{\left(3666n+1858\right)}\\ & - & \frac{\left(3n+6\right)\;\text{log}({\left(37\right)}^{37})}{\left(3666n+1858\right)}-\frac{\left(1\right)\;\text{log}({\left(25\right)}^{25})}{\left(3666n+1858\right)} \end{array}$$

- **First Redefined Zagreb entropy of** *FeCl*_2_

Using Tables 1 and 3 the computed First Redefined Zagreb index is:

$$\begin{array}{c} ReZG_{1}\left(FeCl_{2}\right)=48n+37. \end{array}$$

Using Tables 1–3 we have the following equation because *FeCl*_2_ has nine types of edges:

$$\begin{array}{c} ENT_{ReZG_{1}}\left(K\right)=\text{log}\left(ReZG_{1}\right)-\frac{1}{\left(ReZG_{1}\right)}\sum_{i=1}^{3}\sum_{xy\in E_{i}\left(K\right)}\left(\frac{u^{+}}{u^{*}}\right)\;\text{log}\;\left(\frac{u^{+}}{u^{*}}\right) \end{array}$$

After putting the value of First Redefined Zagreb index and using Table 3 we get.

$$\begin{array}{ccc} ENT_{ReZG_{1}} & = & \text{log}\left(48n+37\right)-\frac{\left(1\right)\;\text{log}({\left(\frac{5}{4}\right)}^{\frac{5}{4}})}{\left(48n+37\right)}-\frac{\left(2\right)\;\text{log}({\left(\frac{6}{5}\right)}^{\frac{6}{5}})}{\left(48n+37\right)}-\frac{\left(3n+6\right)\;\text{log}({\left(\frac{7}{6}\right)}^{\frac{7}{6}})}{\left(48n+37\right)}\\ & - & \frac{\left(2\right)\;\text{log}({\left(\frac{6}{8}\right)}^{\frac{6}{8}})}{\left(48n+37\right)}-\frac{\left(6n+10\right)\;\text{log}({\left(\frac{7}{10}\right)}^{\frac{7}{10}})}{\left(48n+37\right)}-\frac{\left(6n+8\right)\;\text{log}({\left(\frac{8}{12}\right)}^{\frac{8}{12}})}{\left(48n+37\right)}\\ & - & \frac{\left(9n+3\right)\;\text{log}({\left(\frac{8}{15}\right)}^{\frac{8}{15}})}{\left(48n+37\right)}-\frac{\left(63n+10\right)\;\text{log}({\left(\frac{9}{18}\right)}^{\frac{9}{18}})}{\left(48n+37\right)}\\ & - & \frac{\left(1\right)\;\text{log}({\left(\frac{7}{12}\right)}^{\frac{7}{12}})}{\left(48n+37\right)} \end{array}$$

- **Second Redefined Zagreb entropy of** *FeCl*_2_

Using Tables 1 and 3 the computed Second Redefined Zagreb index is:

$$\begin{array}{c} ReZG_{2}\left(FeCl_{2}\right)=163.0178n+82.6512. \end{array}$$

Using Tables 1–3 we have the following equation because *FeCl*_2_ has nine types of edges:

$$\begin{array}{c} ENT_{ReZG_{2}}\left(K\right)=\text{log}\left(ReZG_{2}\right)-\frac{1}{\left(ReZG_{2}\right)}\sum_{i=1}^{m}\sum_{xy\in E_{i}\left(K\right)}\left(\frac{u^{*}}{u^{+}}\right)\;\text{log}\;\left(\frac{u^{*}}{u^{+}}\right) \end{array}$$

After putting the value of Second Redefined Zagreb index and using Table 3 we get.

$$\begin{array}{ccc} ENT_{ReZG_{2}} & = & \text{log}\left(163.0178n+82.6512\right)-\frac{\left(1\right)\;\text{log}({\left(\frac{4}{5}\right)}^{\frac{4}{5}})}{\left(163.0178n+82.6512\right)}\\ & - & \frac{\left(2\right)\;\text{log}({\left(\frac{5}{6}\right)}^{\frac{5}{6}})}{\left(163.0178n+82.6512\right)}-\frac{\left(3n+6\right)\;\text{log}({\left(\frac{6}{7}\right)}^{\frac{6}{7}})}{\left(163.0178n+82.6512\right)}\\ & - & \frac{\left(2\right)\;\text{log}({\left(\frac{8}{6}\right)}^{\frac{8}{6}})}{\left(163.0178n+82.6512\right)}-\frac{\left(6n+10\right)\;\text{log}({\left(\frac{10}{7}\right)}^{\frac{10}{7}})}{\left(163.0178n+82.6512\right)}\\ & - & \frac{\left(6n+8\right)\;\text{log}({\left(\frac{12}{8}\right)}^{\frac{12}{8}})}{\left(163.0178n+82.6512\right)}-\frac{\left(1\right)\;\text{log}({\left(\frac{12}{7}\right)}^{\frac{12}{7}})}{\left(163.0178n+82.6512\right)}\\ & - & \frac{\left(9n+3\right)\;\text{log}({\left(\frac{15}{8}\right)}^{\frac{15}{8}})}{\left(163.0178n+82.6512\right)}-\frac{\left(63n+10\right)\;\text{log}({\left(\frac{18}{9}\right)}^{\frac{18}{9}})}{\left(163.0178n+82.6512\right)} \end{array}$$

- **Third Redefined Zagreb entropy of** *FeCl*_2_

Using Tables 1 and 3 the computed Third Redefined Zagreb index is:

$$\begin{array}{c} ReZG_{3}\left(FeCl_{2}\right)=12408n+5160. \end{array}$$

Using Tables 1–3 we have the following equation because *FeCl*_2_ has nine types of edges:

$$\begin{array}{c} ENT_{ReZG_{3}}\left(K\right)=\text{log}\left(ReZG_{3}\right)-\frac{1}{\left(ReZG_{3}\right)}\sum_{i=1}^{m}\sum_{xy\in E_{i}\left(K\right)}\left(\left(u^{*}\right)\times \left(u^{+}\right)\right)\;\text{log}\;\left(\left(u^{*}\right)\times \left(u^{+}\right)\right) \end{array}$$

After putting value of Third Redefined Zagreb index and using Table 3 we get.

$$\begin{array}{ccc} ENT_{ReZG_{3}} & = & \text{log}\left(12408n+5160\right)-\frac{\left(1\right)\;\text{log}({\left(20\right)}^{20})}{\left(12408n+5160\right)}-\frac{\left(2\right)\;\text{log}({\left(30\right)}^{30})}{\left(12408n+5160\right)}\\ & - & \frac{\left(2\right)\;\text{log}({\left(48\right)}^{48})}{\left(12408n+5160\right)}-\frac{\left(6n+10\right)\;\text{log}({\left(70\right)}^{70})}{\left(12408n+5160\right)}-\frac{\left(6n+8\right)\;\text{log}({\left(96\right)}^{96})}{\left(12408n+5160\right)}\\ & - & \frac{\left(9n+3\right)\;\text{log}({\left(120\right)}^{120})}{\left(12408n+5160\right)}-\frac{\left(63n+10\right)\;\text{log}({\left(162\right)}^{162})}{\left(12408n+5160\right)}\\ & - & \frac{\left(3n+6\right)\;\text{log}({\left(42\right)}^{42})}{\left(12408n+5160\right)}-\frac{\left(1\right)\;\text{log}({\left(84\right)}^{84})}{\left(12408n+5160\right)} \end{array}$$

**Fig 6:**
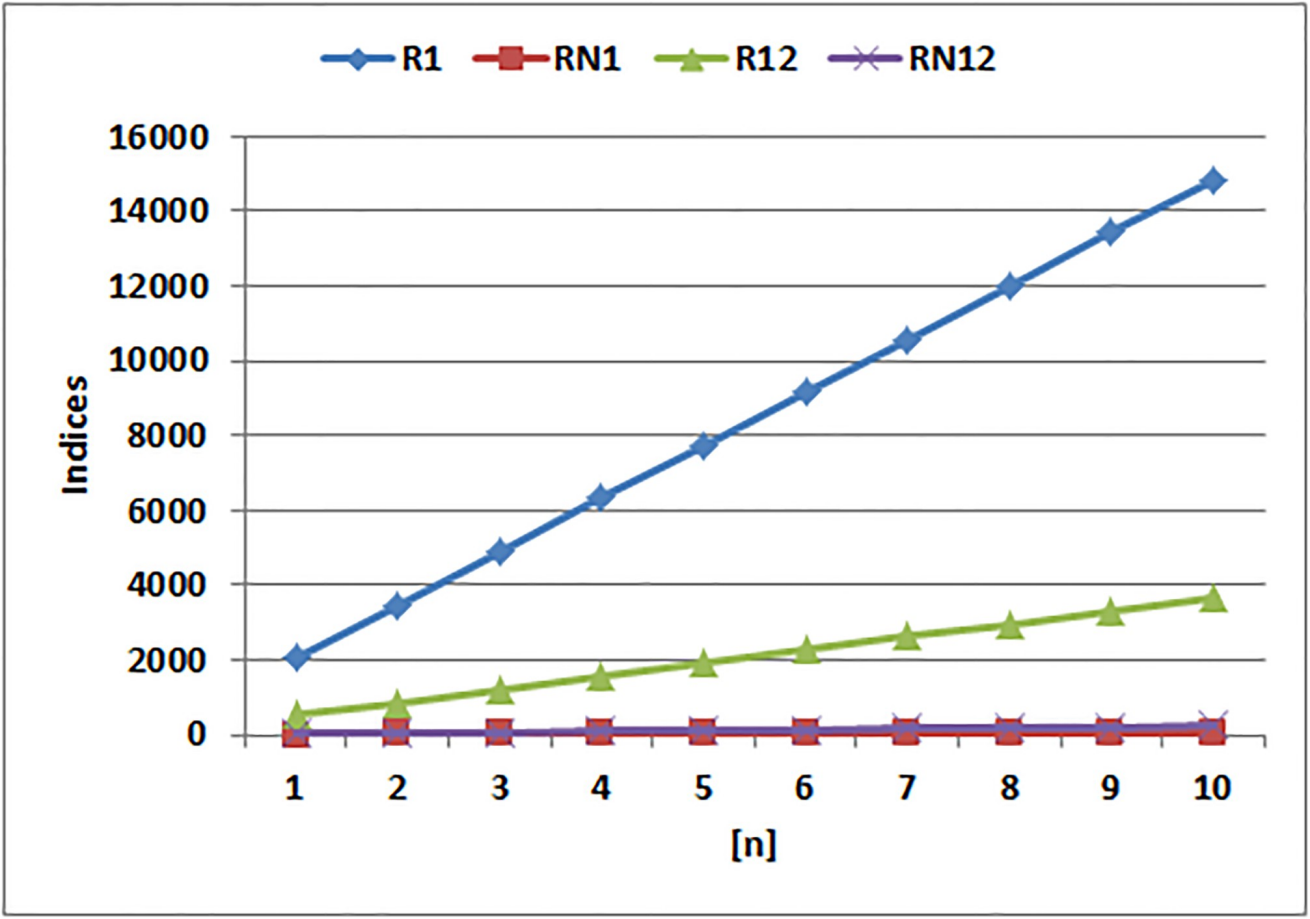
Graphical comparison of Randic indices.

**Fig 7:**
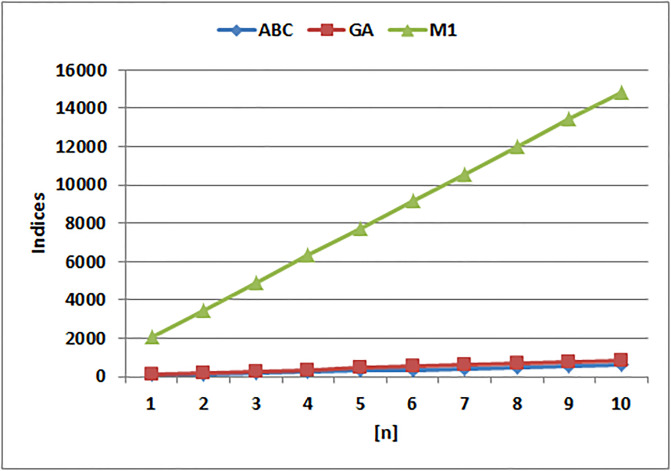
Graphical comparison of *ABC*, *GA*, *M*1.

**Fig 8:**
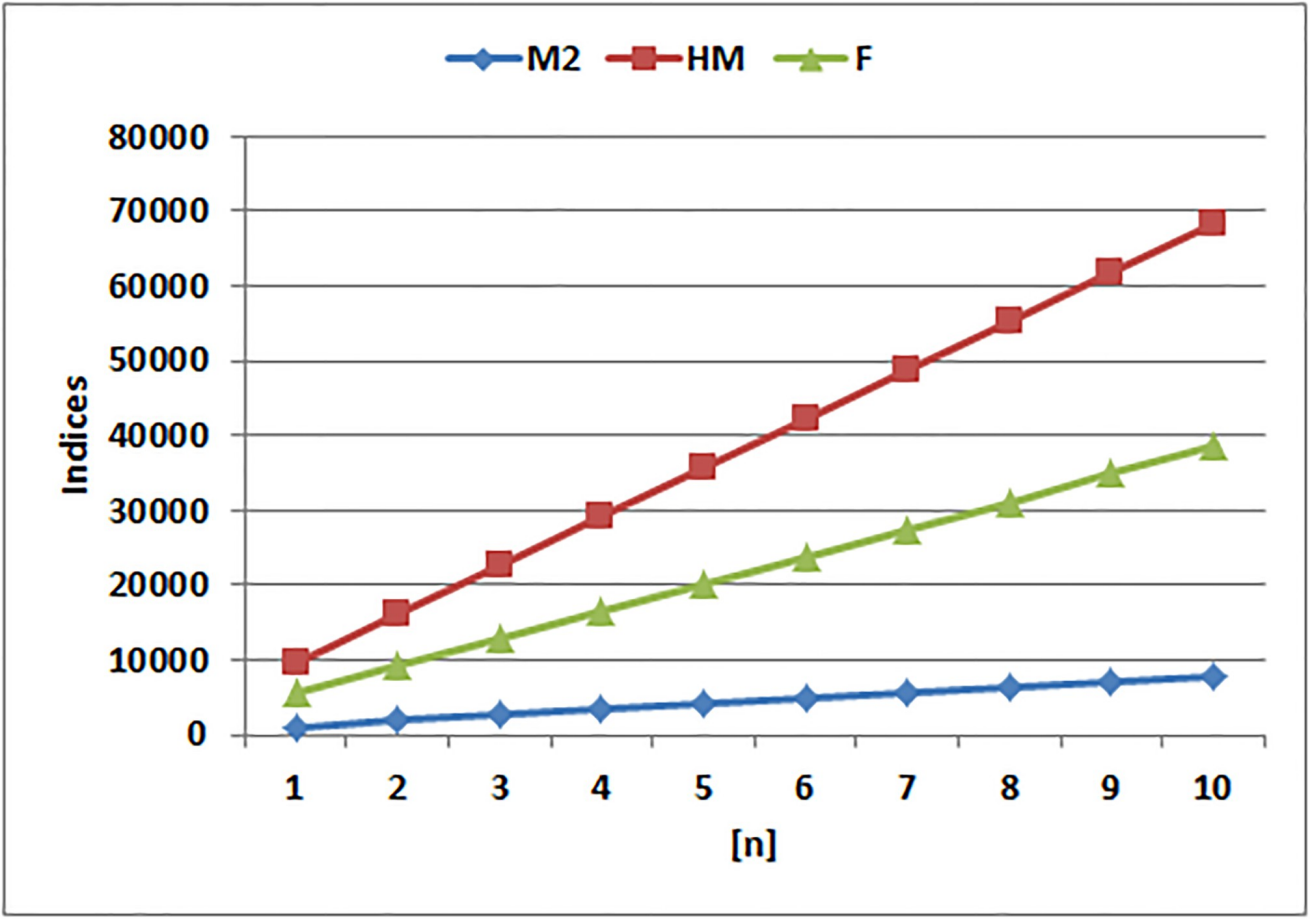
Graphical comparision of *M*_2_, *HM*, *F*.

**Fig 9:**
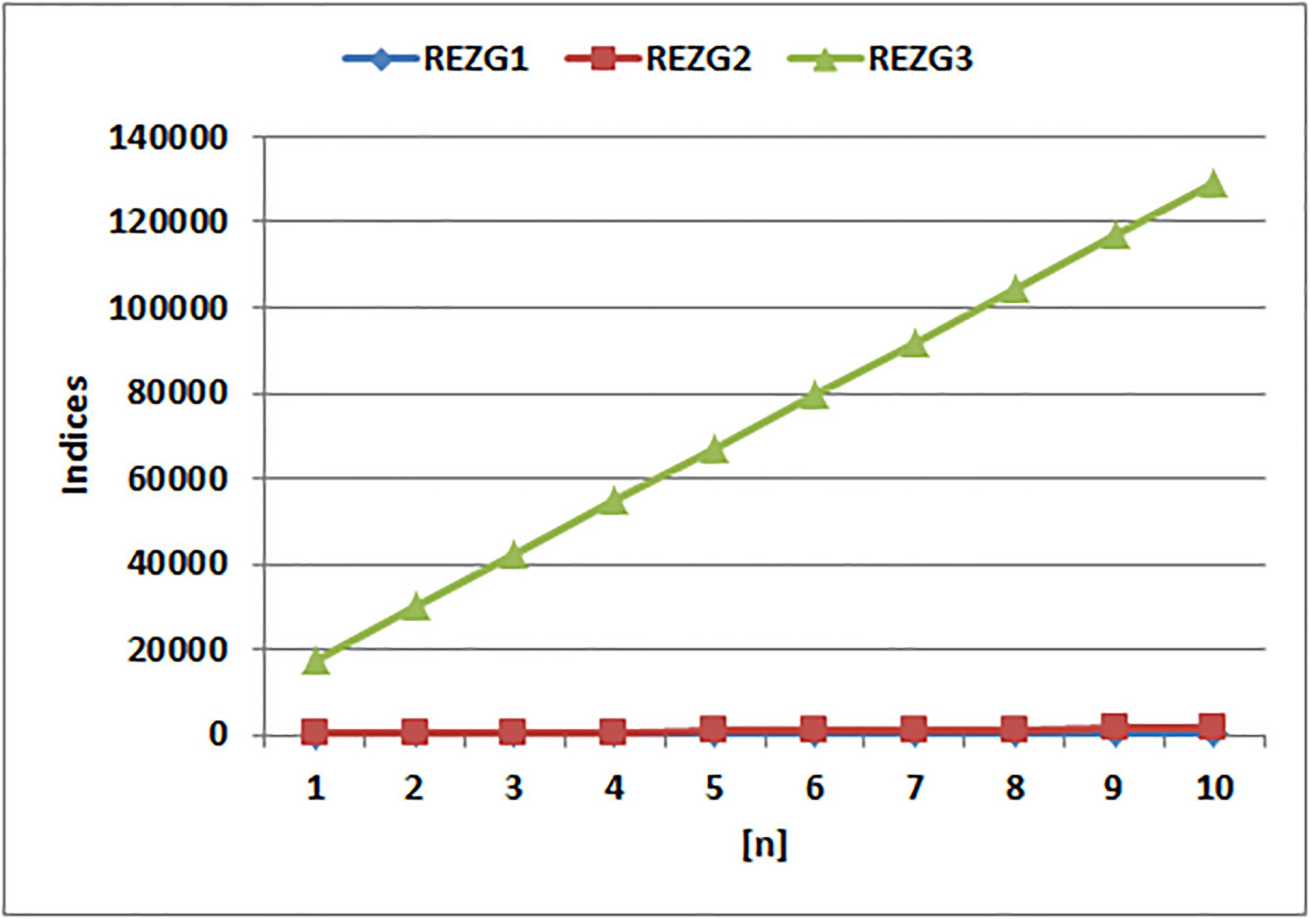
To aid with visual comprehension.

Table 4 presents the numerical values of degree-based indices, while Table 5 displays the corresponding entropy values. These tables provide a comprehensive overview of the calculated indices and their associated entropy measures, offering quantitative insights into the structural characteristics and complexity of the analyzed molecules. Graphical depictions of the data are shown in Figs 6–9 to aid with visual comprehension. These diagrams give the links between the degree-based indices and entropy a visual representation, making it easier to understand the data. The examination and interpretation of the results are aided by the graphical representations, which make any trends, patterns, or correlations between the indices and entropy measures easily visible.

**Table 4:** Degree-based topological indices of iron (II) chloride.

Indices/[n]	[1]	[2]	[3]	[4]	[5]	[6]	[7]	[8]	[9]	[10]
*R*_1_(*K*)	2068	3487	4906	6325	7744	9163	10582	12001	13420	14839
*R*_−1_(*K*)	10.77	16.47	22.17	27.87	33.57	39.27	44.97	50.67	56.37	62.07
$R_{\frac{1}{2}}(K)$	531.65	880.90	1230.15	1579.40	1928.65	2277.90	2627.15	2976.40	3325.64	3674.89
$R_{-\frac{1}{2}}(K)$	38.05	60.07	82.12	104.13	126.16	148.18	170.22	192.24	214.26	236.29
*ABC*(*K*)	93.58	149.78	205.98	262.19	318.39	374.59	430.80	487.00	543.20	599.41
*GA*(*K*)	128.17	208.99	289.82	370.65	451.48	532.31	613.13	693.96	774.79	855.62
*M*_1_(*K*)	1156	1906	2656	3406	4156	4906	5656	6406	7156	7906
*M*_2_(*K*)	2068	3487	4906	6325	7744	9163	10582	12001	13420	14839
*HM*(*K*)	9660	16164	22668	29172	35676	42180	48684	55188	61692	68196
*F*(*K*)	5524	9190	12856	16522	20188	23854	27520	31186	34852	38518
*ReZG*_1_(*K*)	85	133	181	229	277	325	373	421	469	517
*ReZG*_2_(*K*)	245.669	408.68	571.70	734.72	897.74	1060.75	1223.77	1386.79	1549.81	1712.82
*ReZG*_3_(*K*)	17568	29976	42384	54792	67200	79608	92016	104424	116832	129240

**Table 5:** Degree-based entropy of iron (II) chloride.

Entropy/[n]	[1]	[2]	[3]	[4]	[5]	[6]	[7]	[8]	[9]	[10]
$ENT_{R_{1}}(FeCl_{2})$	4.8978	5.3889	5.7171	5.9638	6.1616	6.3266	6.4683	6.5923	6.7027	6.8021
$ENT_{R_{-1}}(FeCl_{2})$	4.1164	4.6684	5.0274	5.2927	5.5029	5.6769	5.8252	5.9544	6.0689	6.1717
$ENT_{R_{\frac{1}{2}}}(FeCl_{2})$	4.9285	5.4140	5.7395	5.9848	6.1815	6.3459	6.4870	6.6107	6.7207	6.8198
$ENT_{R_{-\frac{1}{2}}}(FeCl_{2})$	4.9293	5.4129	5.7377	5.9826	6.1791	6.3434	6.4844	6.6079	6.7179	6.8170
*ENT*_*ABC*_(*FeCl*_2_)	4.9349	5.4192	5.7442	5.9890	6.1856	6.3498	6.4908	6.6144	6.7243	6.8234
*ENT*_*GA*_(*FeCl*_2_)	4.9387	5.4225	5.7472	5.9919	6.1883	6.3524	6.4934	6.6169	6.7268	6.8258
$ENT_{M_{1}}(FeCl_{2})$	4.9353	5.4197	5.7446	5.9895	6.1861	6.3503	6.4913	6.6149	6.7248	6.8239
$ENT_{M_{2}}(FeCl_{2})$	4.8978	5.3889	5.7171	5.9638	6.1616	6.3266	6.4683	6.5923	6.7027	6.8021
*ENT*_*HM*_(*FeCl*_2_)	4.9189	5.4060	5.7324	5.9781	6.1752	6.3398	6.4811	6.6049	6.7150	6.8142
*ENT*_*F*_(*FeCl*_2_)	4.9254	5.4116	5.7374	5.9828	6.1797	6.3442	6.4854	6.6091	6.7191	6.8183
$ENT_{ReZG_{1}}(FeCl_{2})$	4.8998	5.3888	5.7163	5.9628	6.1604	6.3254	6.4670	6.5910	6.7013	6.8007
$ENT_{ReZG_{2}}(FeCl_{2})$	4.9185	5.4059	5.7323	5.9780	6.1751	6.3397	6.4810	6.6048	6.7149	6.8141
$ENT_{ReZG_{3}}(FeCl_{2})$	4.8718	5.3676	5.6980	5.9460	6.1446	6.3103	6.4524	6.5767	6.6874	6.7870

Fig 6 shows that, in contrast to the Randic indices, the Randic index for *alpha* = 1 exhibits a sharp increase. The Randic index values for the *alpha* = 1 clearly show a sharp increasing trend in the graph, showing a significant increase in branching and complexity. The other Randic indices, however, show considerably slower rates.

The *ABC*(*G*), *GA*(*G*), and *M*_1_(*G*) indices all exhibit distinct patterns of change, as is clear from Fig 7. When compared to the other indices, the *M*_1_(*G*) index shows a substantially more rapid increase, it becomes clear from a closer look. The *M*_1_(*G*) index values rise significantly as the graph develops, showing a rapid increase in complexity and structural variety. The *GA*(*G*) and *ABC*(*G*), indices, on the other hand, exhibit comparatively slower rates of rise, which suggests that complexity and variety have accumulated more slowly overall. This finding emphasizes how important the *M*_1_(*G*) index is for capturing the complex traits and connection patterns inside a given molecular structure.

It is clear from examining the Fig 8 that the *M*_2_(*G*), *HM*(*G*), and *F*(*G*) indices display unique trends. In particular, the *HM*(*G*) index shows a quick rise in comparison to the other indices. The *HM*(*G*) index values grow sharply, as seen in the graph, indicating a rapid development in complexity and structural variety within the molecular system under investigation. The *M*_2_(*G*) and *F*(*G*) indices, on the other hand, exhibit somewhat slower rates of rise, pointing to a more gradual buildup of complexity and diversity. The *HM*(*G*) index has had a strong rise, which emphasizes its special sensitivity in capturing the complex features and connection patterns within the provided molecular structure.

It is clear from Fig 9 that the Redefined Zagreb indices show various patterns of change. Notably, the Third redefined Zagreb index stands out from the others because it exhibits a substantial increase. The Third Zagreb index values climb significantly, as seen in the graph, showing a rapid buildup of structural complexity and connection inside the molecule. The other Zagreb indices, on the other hand, exhibit comparably slower rates of increase, pointing to a slower increase in complexity. The Third redefined Zagreb index has grown significantly and quickly, highlighting its capacity to capture and measure the complex structural properties of the molecule under study.

**Fig 10:**
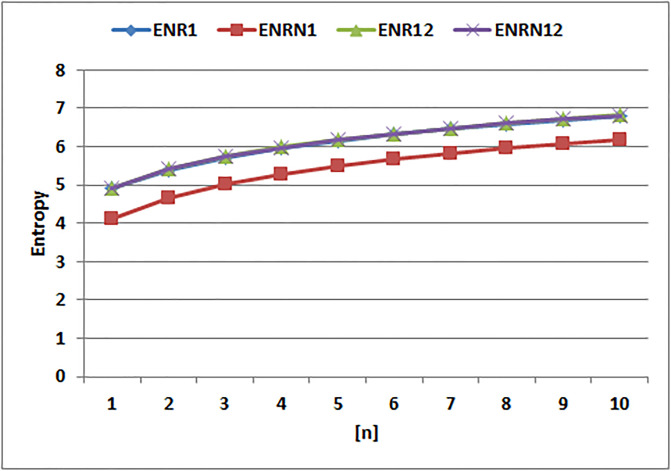
Graphical comparison of Randic entropies.

Fig 10 makes it clear that there are various patterns of change in the entropy values for the Randic indices. Notably, compared to the entropies of other Randic indices, the Randic index for $\alpha =-\frac{1}{2}$ exhibits a quick increase in entropy. The entropy for $\alpha =-\frac{1}{2}$ increases sharply, as shown in the graph, showing a notable rise in the complexity and unpredictability of the related molecular structures. The other entropy of Randic indices, on the other hand, exhibits slower rates of development, pointing to a more gradual increase in complexity and unpredictability. The Randic index for $\alpha =-\frac{1}{2}$ dramatic increase in entropy draws attention to how sensitive it is to capture the various structural traits and patterns found in the molecules under study.

**Fig 11:**
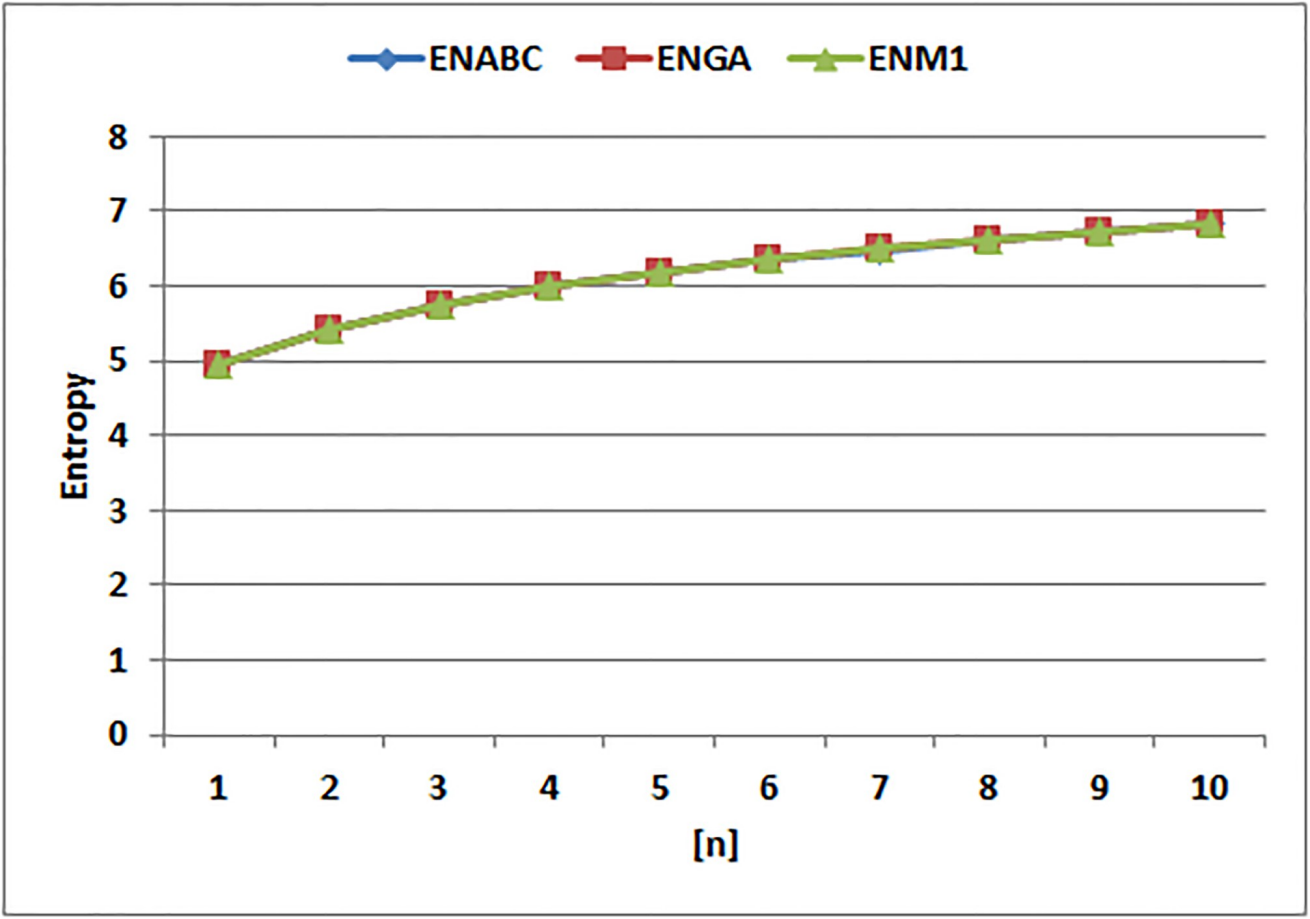
Graphical comparision of *ENABC*, *ENGA*, *ENM*1.

The entropy values for *EABC*(*G*), *EGA*(*G*), and *EM*_1_(*G*) display distinct tendencies, as can be seen by looking at Fig 11. Particularly, the entropy of *EM*_1_(*G*) stands out since, when compared to the other entropy values, it shows a quick increase. The entropy values for *EM*_1_(*G*) climb sharply, as seen in the graph, indicating a notable increase in complexity and unpredictability inside the related molecular structures. The entropies of *EGA*(*G*) and *EABC*(*G*), on the other hand, exhibit considerably slower rates of rise, indicating a more gradual buildup of complexity and randomness. The significant and quick increase in entropy of *EM*_1_(*G*) demonstrates how well it captures and measures the complex traits and variety present in the examined molecular systems.

**Fig 12:**
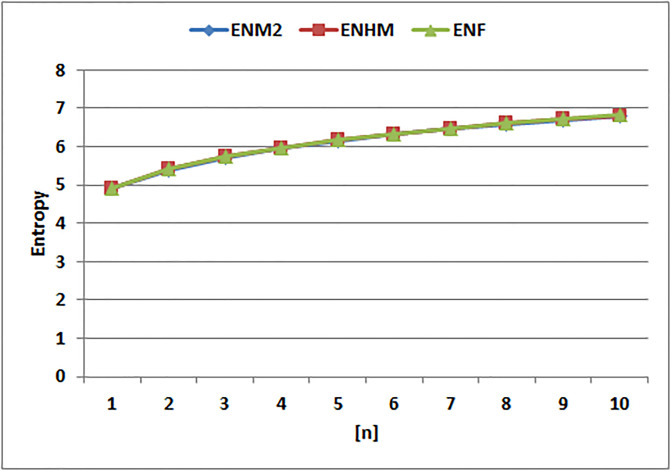
Graphical comparision of *ENM*2, *ENHM*, *ENF*.

The entropy values for *EM*_2_(*G*), *EHM*(*G*), and *EF*(*G*) clearly show separate patterns of change, as shown in Fig 12. Notably, when compared to the entropy of the other indices, the entropy of *EF*(*G*) stands out since it increases quickly. The molecular systems’ complexity and unpredictability have grown significantly over time, as shown by the graph’s sharp rising trend in entropy values for *EF*(*G*). The entropy’s of *EM*_2_(*G*) and *EHM*(*G*), on the other hand, exhibit comparatively slower rates of rise, pointing to a more gradual buildup of complexity and randomness. The *EF*(*G*) ability to capture and quantify the many structural properties and information content inside the studied molecule structures is highlighted by the sharp increase in entropy.

**Fig 13:**
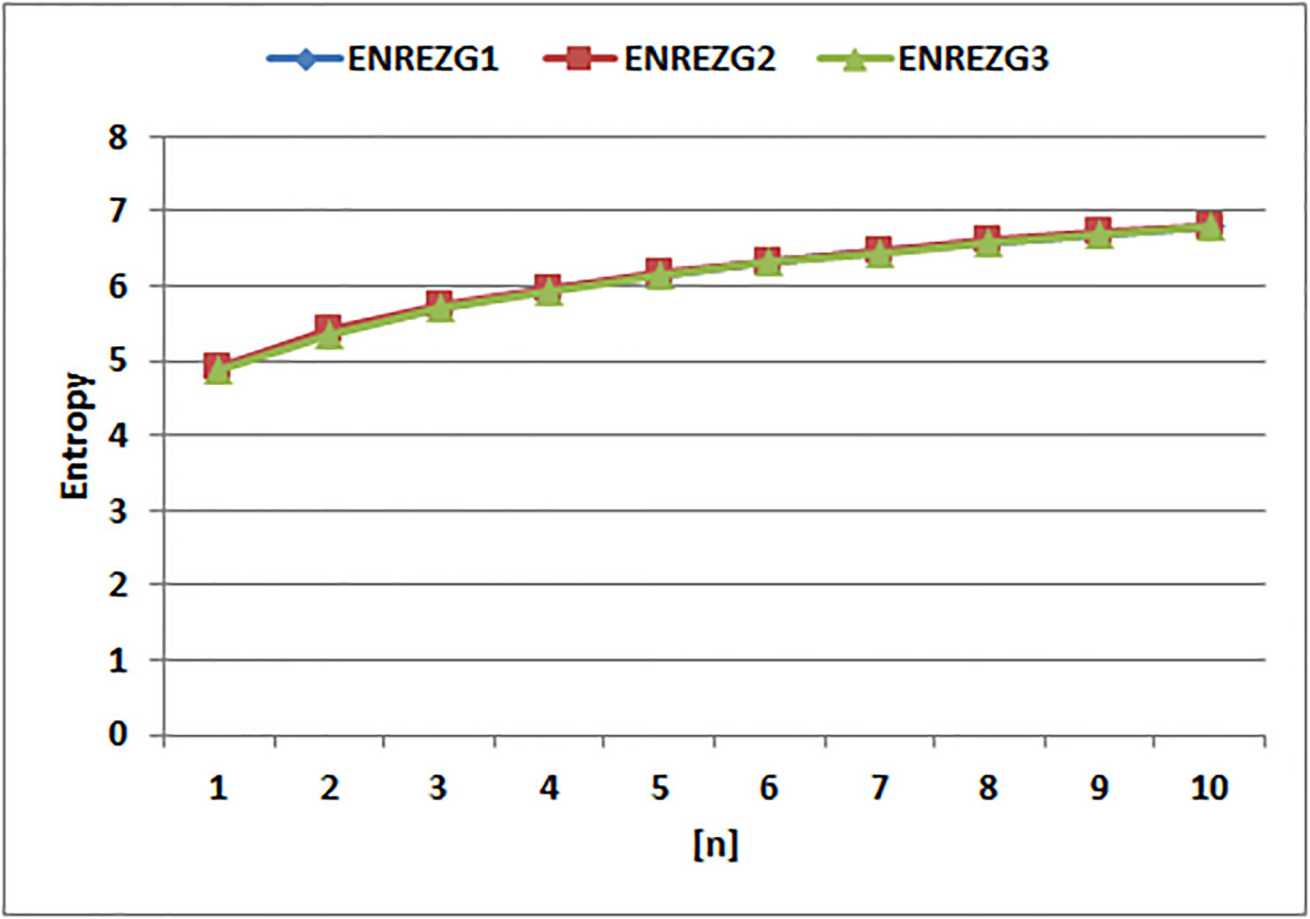
Graphical comparision of Redefined Zagreb entropies.

It is clear from looking at Fig 13 that the redefined Zagreb indices entropy values exhibit unique tendencies. To be more precise, in contrast to the entropy of the previous indices, the second redefined Zagreb index entropy is seen to be fast-rising. The graph shows a sharp increase in entropy values for the second redefined Zagreb index, which is an indication of significant development in complexity and randomness within the related molecular structures. The other redefined Zagreb indices entropies, on the other hand, exhibit comparatively slower rates of growth, which suggests a more gradual buildup of complexity and randomness. The second revised Zagreb index has had a considerable and quick growth in entropy, highlighting its capacity to capture and measure the many structural aspects and information content within the studied molecular systems.

## 5. Exploring the relationship between indices and entropy via correlation analysis

In this section, we investigate the relationship between degree-based topological indices and entropy measurements. These correlations have important applications in a number of fields. For a variety of distinct values of n, the entropies for *FeCl*_2_ were painstakingly computed. Researchers can learn a great deal about the structural properties and information content of the *FeCl*_2_ molecules by looking at these entropies, enabling additional investigation and analysis in a variety of scientific domains. Additionally, Table 4 represent the values of degree-based topological indices, while Table 5 represent the entropy formation of *FeCl*_2_ structures.

The line fitting results, illustrating the correlation between degree-based indices and entropy measures, are presented in the analysis as shown in Figs 14–26.

**Fig 14:**
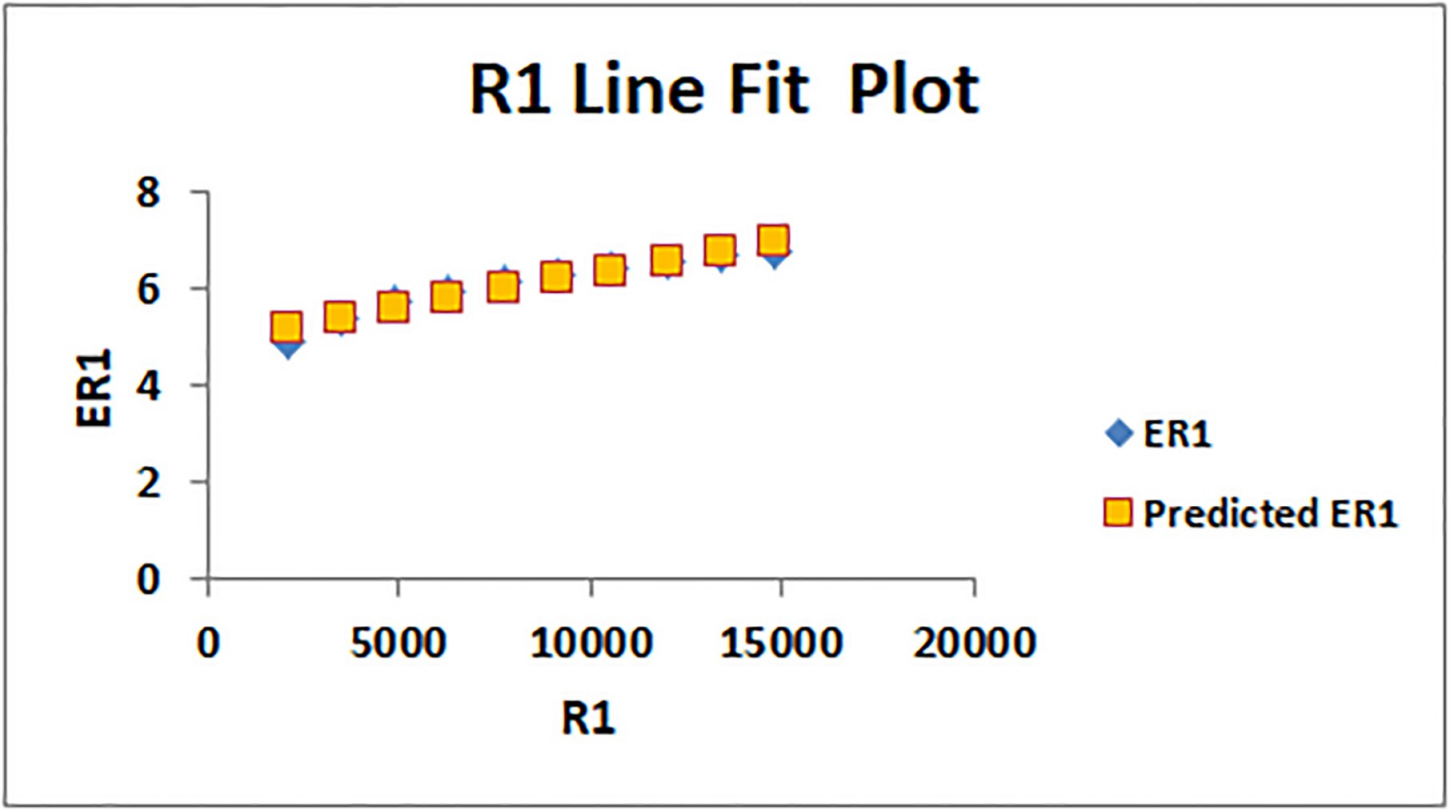
Analysis correlation between indices and entropy measures *R*1, *ER*1.

**Fig 15:**
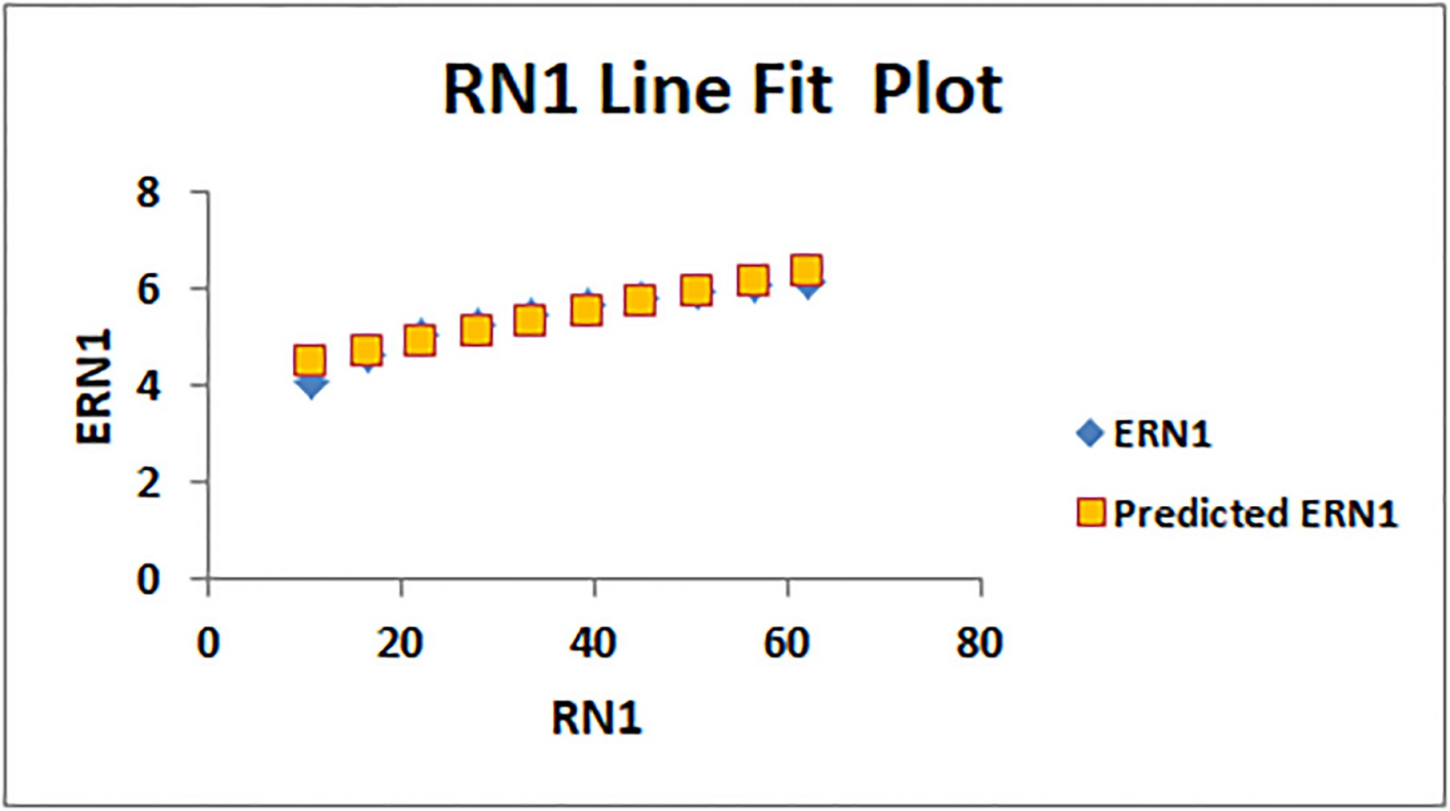
Analysis correlation between indices and entropy measures *RN*1, *ERN*1.

**Fig 16:**
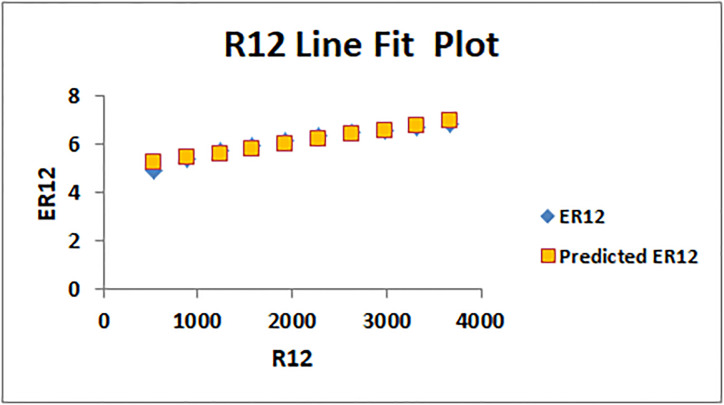
Analysis correlation between indices and entropy measures (*a*)*R*12, *ER*12.

**Fig 17:**
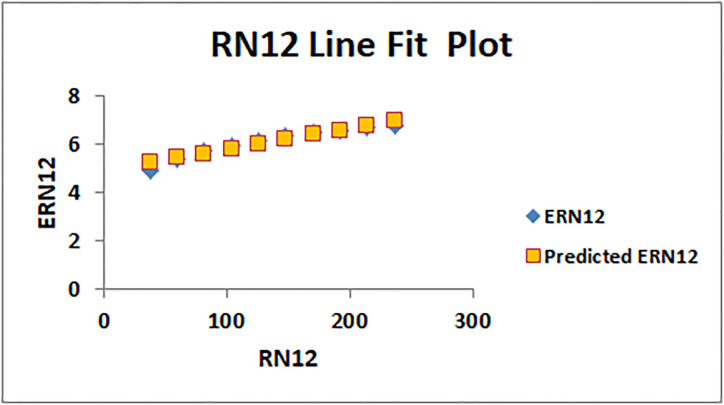
Analysis correlation between indices and entropy measures *RN*12, *ERN*12.

**Fig 18:**
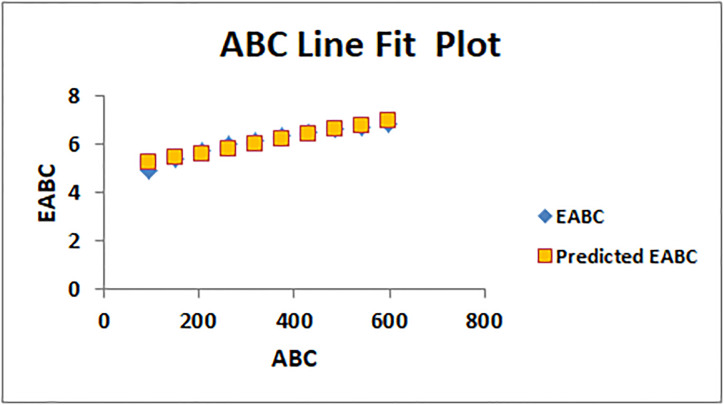
Analysis correlation between indices and entropy measures (*a*)*ABC*, *EABC*.

**Fig 19:**
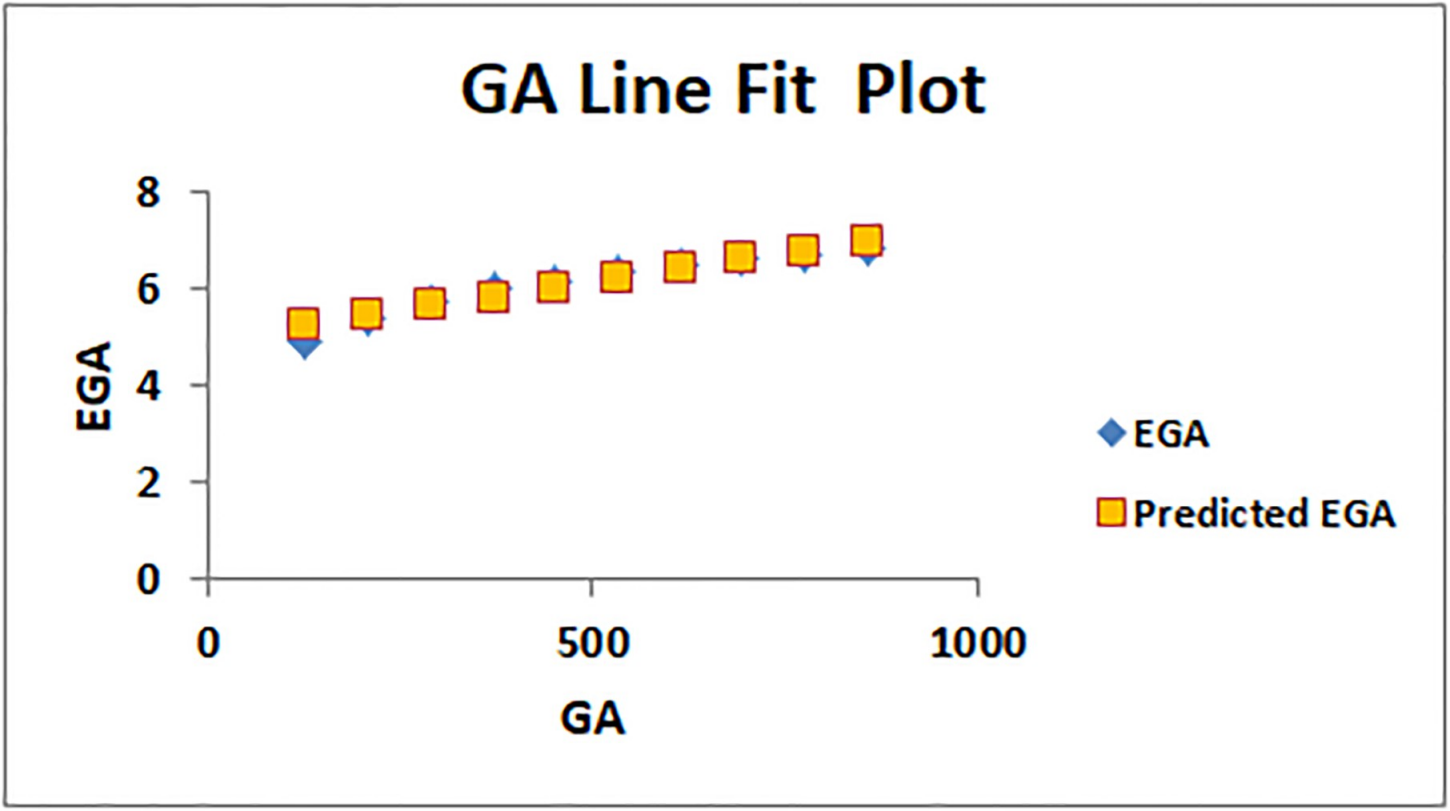
Analysis correlation between indices and entropy measures *GA*, *EGA*.

**Fig 20:**
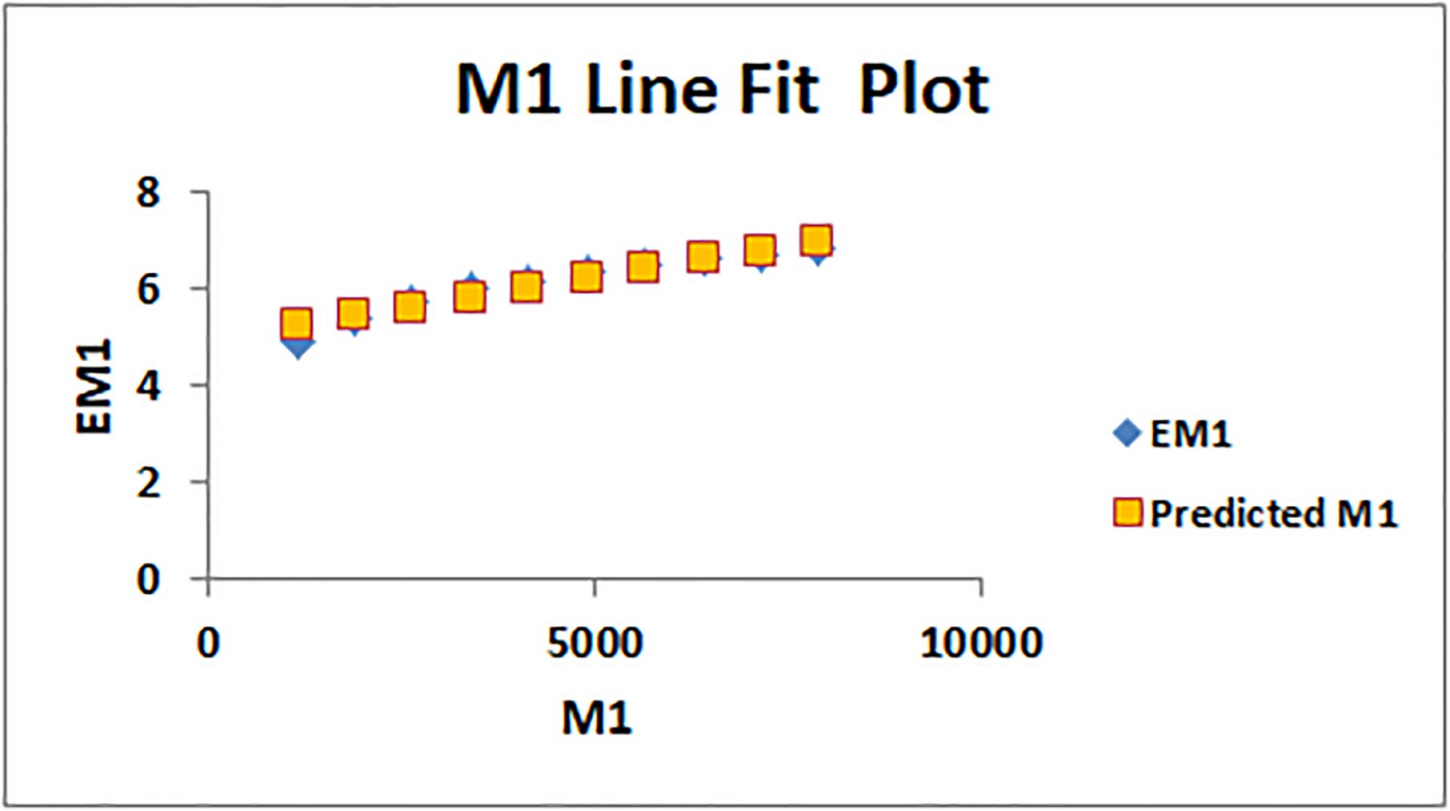
Analysis correlation between indices and entropy measures *M*1, *EM*1.

**Fig 21:**
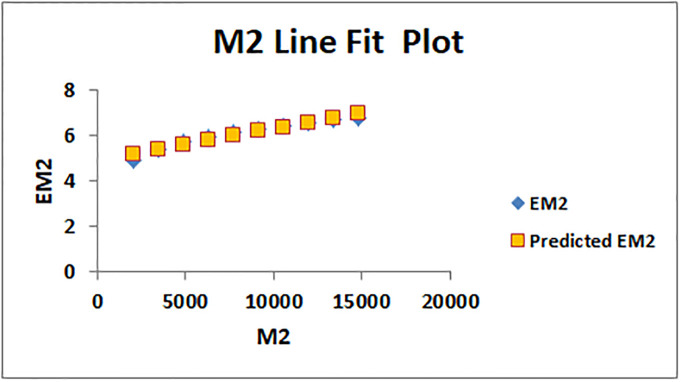
Analysis correlation between indices and entropy measures *M*2, *EM*2.

**Fig 22:**
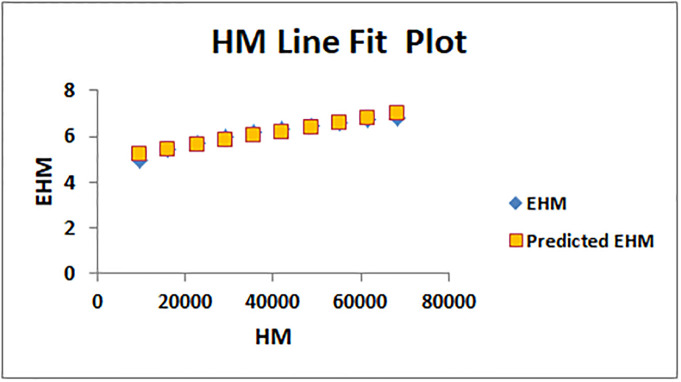
Analysis correlation between indices and entropy measures *HM*, *EHM*.

**Fig 23:**
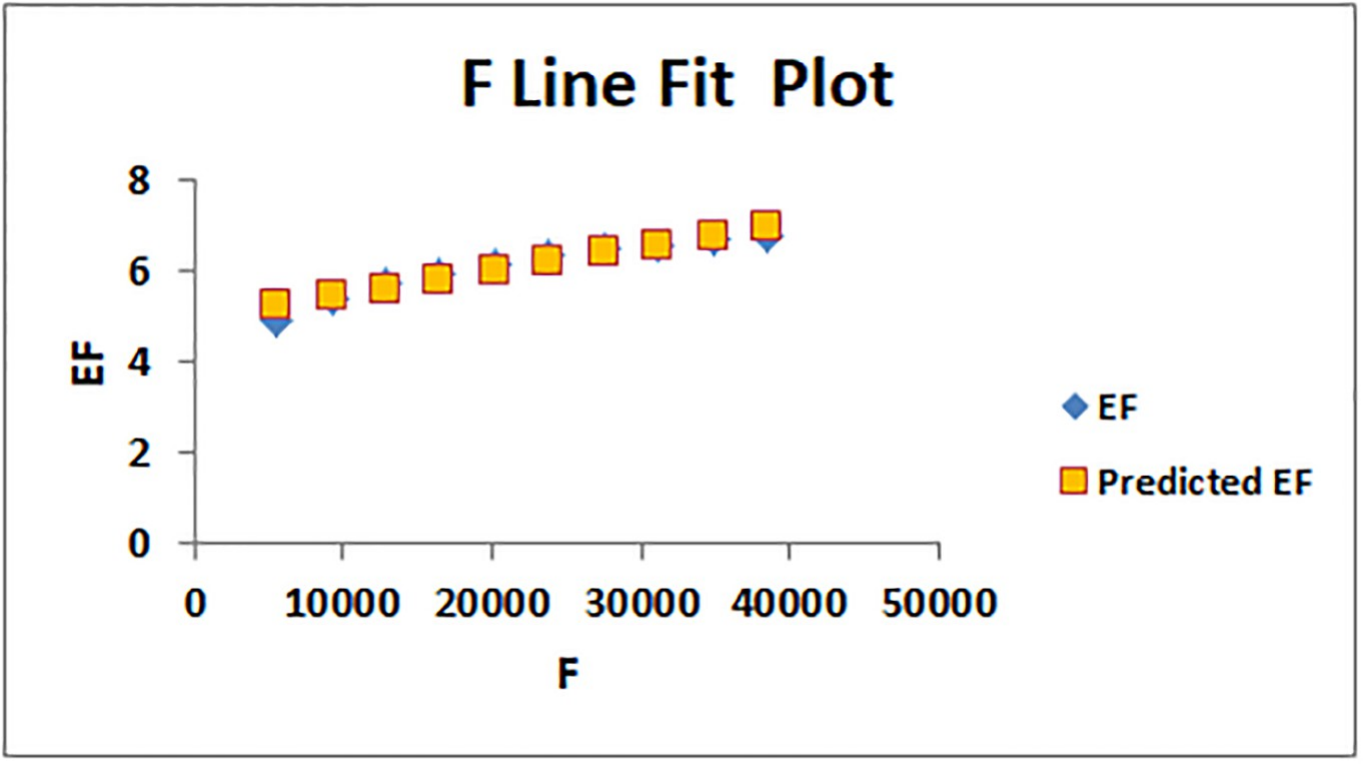
Analysis correlation between indices and entropy measures *F*, *EF*.

**Fig 24:**
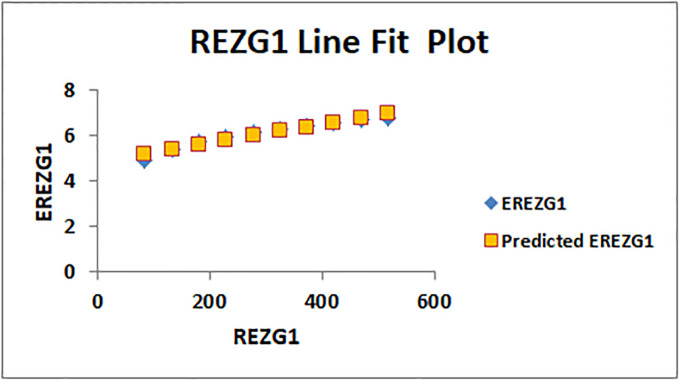
Analysis correlation between indices and entropy measures *REZG*1, *EREZG*1.

**Fig 25:**
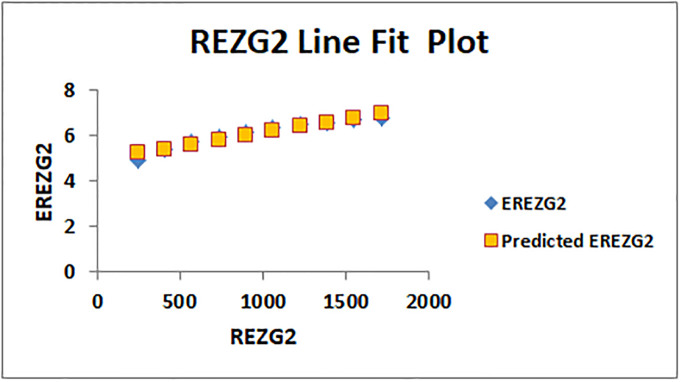
Analysis correlation between indices and entropy measures *REZG*2, *EREZG*2.

**Fig 26:**
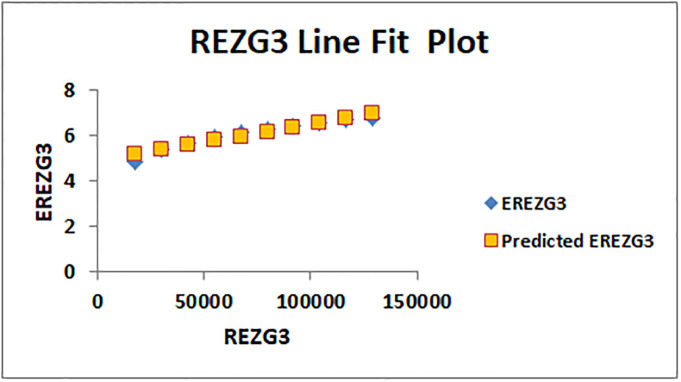
Analysis correlation between indices and entropy measures *REZG*_3_, *EREZG*_3_.

This method of line fitting enables a thorough investigation of the relationship between several variables. The goal of this study was to determine how entropy formation and various indices relate to one another. A line-fitting method was used to adjust underlying parameters in order to find the best fit between entropy and all of the taken-into-consideration indices. The linear regression approach, standard error estimation, *R*, *R*^2^, and the coefficients *a* and *b* were used to assess the accuracy of the fit. To evaluate the significance of the association, special attention was paid to the *R*^2^ value. All simulations were conducted using Excel. To further assess the values shown in Table 6 presents the correlation coefficient of the indices in relation to entropy. Additionally, Table 7 displays the predicted entropies based on the curve fitting results.

**Table 6:** Linear regression models and coefficients for the relationship between indices.

Indices	a	b	R	*R* ^2^	SE
*R*_1_(*FeCl*_2_)	0.000138338	4.932678548	0.966199878	0.933542204	0.168194694
*R*_−1_(*FeCl*_2_)	0.036903456	4.086384959	0.962266017	0.925955888	0.191017866
$R_{\frac{1}{2}}(FeCl_{2})$	0.000558697	4.948146566	0.966599142	0.934313902	0.166144086
$R_{-\frac{1}{2}}(FeCl_{2})$	0.008844437	4.907988782	0.966750726	0.934606966	0.165486551
*ABC*(*FeCl*_2_)	0.003467153	4.926205786	0.966685048	0.934479981	0.165698601
*GA*(*FeCl*_2_)	0.002409242	4.945290681	0.966714227	0.934536396	0.165510471
*M*_1_(*FeCl*_2_)	0.000259832	4.950741574	0.966681692	0.934473494	0.165714594
*M*_2_(*FeCl*_2_)	0.000138338	4.932678548	0.966199878	0.933542204	0.168194694
*HM*(*FeCl*_2_)	3.00578*E* − 05	4.946471001	0.966484277	0.934091858	0.166760835
*F*(*FeCl*_2_)	5.32649*E* − 05	4.948353832	0.966553946	0.934226531	0.166385226
*ReZG*_1_(*FeCl*_2_)	0.004084179	4.872012033	0.966379833	0.933889983	0.167499608
*ReZG*_2_(*FeCl*_2_)	0.00119933	4.941987278	0.966462296	0.93404937	0.166832335
*ReZG*_3_(*FeCl*_2_)	1.59007*E* − 05	4.917005775	0.965866182	0.932897481	0.169923004

**Table 7:** Predicted entropy.

Predicted entropy	[1]	[2]	[3]	[4]	[5]	[6]	[7]	[8]	[9]	[10]
$ENT_{R_{1}}(FeCl_{2})$	5.2187	5.4150	5.6113	5.8076	6.0039	6.2002	6.3965	6.5928	6.7891	6.9854
$ENT_{R_{-1}}(FeCl_{2})$	4.4839	4.6942	4.9046	5.1149	5.3253	5.5356	5.7460	5.9563	6.1667	6.3770
$ENT_{R_{\frac{1}{2}}}(FeCl_{2})$	5.2451	5.4403	5.6354	5.8305	6.0256	6.2208	6.4159	6.6110	6.8061	7.0013
$ENT_{R_{-\frac{1}{2}}}(FeCl_{2})$	5.2445	5.4393	5.6341	5.8289	6.0238	6.2186	6.4134	6.6082	6.8030	6.9979
*ENT*_*ABC*_(*FeCl*_2_)	5.2506	5.4455	5.6403	5.8352	6.0301	6.2249	6.4198	6.6147	6.8095	7.0044
*ENT*_*GA*_(*FeCl*_2_)	5.2540	5.4488	5.6435	5.8382	6.0330	6.2277	6.4224	6.6172	6.8119	7.0066
$ENT_{M_{1}}(FeCl_{2})$	5.2511	5.4459	5.6408	5.8357	6.0306	6.2254	6.4203	6.6152	6.8100	7.0049
$ENT_{M_{2}}(FeCl_{2})$	5.2187	5.4150	5.6113	5.8076	6.0039	6.2002	6.3965	6.5928	6.7891	6.9854
*ENT*_*HM*_(*FeCl*_2_)	5.2368	5.4323	5.6278	5.8233	6.0188	6.2143	6.4098	6.6052	6.8007	6.9962
*ENT*_*F*_(*FeCl*_2_)	5.2425	5.4378	5.6331	5.8283	6.0236	6.2189	6.4142	6.6094	6.8047	7.0000
$ENT_{ReZG_{1}}(FeCl_{2})$	5.2191	5.4152	5.6112	5.8072	6.0033	6.1993	6.3954	6.5914	6.7874	6.9835
$ENT_{ReZG_{2}}(FeCl_{2})$	5.2366	5.4321	5.6276	5.8231	6.0186	6.2141	6.4096	6.6052	6.8007	6.9962
$ENT_{ReZG_{3}}(FeCl_{2})$	5.1963	5.3936	5.5909	5.7882	5.9855	6.1828	6.3801	6.5774	6.7747	6.9720

Here are the linear regression models corresponding to each index and entropy.

$$\begin{array}{c} ENT_{R_{1}}\left(K\right)=0.000138338\left[R_{1}\left(K\right)\right]+4.932678548 \end{array}$$ (2)

$$\begin{array}{c} ENT_{R_{-1}}\left(K\right)=0.036903456\left[R_{-1}\left(K\right)\right]+4.086384959 \end{array}$$ (3)

$$\begin{array}{c} ENT_{R_{\frac{1}{2}}}\left(K\right)=0.000558697\left[R_{\frac{1}{2}}\left(K\right)\right]+4.948146566 \end{array}$$ (4)

$$\begin{array}{c} ENT_{R_{-\frac{1}{2}}}\left(K\right)=0.008844437\left[R_{-\frac{1}{2}}\left(K\right)\right]+4.9079887 \end{array}$$ (5)

$$\begin{array}{c} ENT_{ABC}\left(K\right)=0.003467153\left[ABC\left(K\right)\right]+4.9262057 \end{array}$$ (6)

$$\begin{array}{c} ENT_{GA}\left(K\right)=0.002409242\left[GA\left(K\right)\right]+4.945290681 \end{array}$$ (7)

$$\begin{array}{c} ENT_{M_{1}}\left(K\right)=0.000259832\left[M_{1}\left(K\right)\right]+4.950741574 \end{array}$$ (8)

$$\begin{array}{c} ENT_{M_{2}}\left(K\right)=0.000138338\left[M_{2}\left(K\right)\right]+4.932678548 \end{array}$$ (9)

$$\begin{array}{c} ENT_{HM}\left(K\right)=3.00578E-05\left[HM\left(K\right)\right]+4.9464710 \end{array}$$ (10)

$$\begin{array}{c} ENT_{F}\left(K\right)=5.32649E-05\left[F\left(K\right)\right]+4.948353832 \end{array}$$ (11)

$$\begin{array}{c} ENT_{ReZG_{1}}\left(K\right)=0.004084179\left[ReZG_{1}\left(K\right)\right]+4.8720 \end{array}$$ (12)

$$\begin{array}{c} ENT_{ReZG_{2}}\left(K\right)=0.00119933\left[ReZG_{2}\left(K\right)\right]+4.94198 \end{array}$$ (13)

$$\begin{array}{c} ENT_{ReZG_{3}}\left(K\right)=1.59007E-05\left[ReZG_{3}\left(K\right)\right]+4.91 \end{array}$$ (14)

The predicted entropy is shown in Table 7 which is obtained by using linear regression and line fit method.

## 6. Conclusion

In conclusion, the main goal of our research has been to study the entropy of *FeCl*_2_ molecules using degree-based topological descriptors. We have learned a lot about how entropy and different indices relate by generalising analytical formulations and looking into different structural elements. The results show that the entropy changes most significantly in the transition state *FeCl*_2_ as the disorder rises, and the Randic index $\gamma =-\frac{1}{2}$ provides the most consistent and dependable results, as shown by its high *R*^2^ value. This topological descriptor-based mathematical approach offers a thorough grasp of the significant thermodynamic parameters, particularly entropy, that may help direct structural alterations for particular *FeCl*_2_ molecule applications. Additionally, calculating degree-based topological indices provides a framework for integrating metrics from quantum chemistry properties like polarizability, atomic charges, and molecular hardness, which also encourage continued development in the subject. We have investigated the relationship between entropy formation and various indices using line fitting methods and have provided both numerical and graphical evidence to support our results. This study adds to the body of knowledge about entropy and how it affects molecular systems.
